# Impact of sympathetic hyperactivity induced by brain microglial activation on organ damage in sepsis with chronic kidney disease

**DOI:** 10.1186/s40560-024-00742-2

**Published:** 2024-09-02

**Authors:** Masaaki Nishihara, Keisuke Shinohara, Shota Ikeda, Tomohiko Akahoshi, Hiroyuki Tsutsui

**Affiliations:** 1 Emergency and Critical Care Center, Kyushu University Hospital, Fukuoka, Japan; 2 Department of Cardiovascular Medicine, Faculty of Medical Sciences, Kyushu University, Fukuoka, Japan; 3 Department of Advanced Emergency and Disaster medicine, Graduate School of Medical Sciences, Kyushu University, Fukuoka, Japan; 4 School of Medicine and Graduate School, International University of Health and Welfare, Fukuoka, Japan

**Keywords:** Sepsis, Chronic kidney disease, Sympathetic nerve activity, Paraventricular nucleus of the hypothalamus, Microglia

## Abstract

**Background:**

Sympathetic nerve activity (SNA) plays a central role in the pathogenesis of several diseases such as sepsis and chronic kidney disease (CKD). Activation of microglia in the paraventricular nucleus of the hypothalamus (PVN) has been implicated in SNA. The mechanisms responsible for the adverse prognosis observed in sepsis associated with CKD remain to be determined. Therefore, we aimed to clarify the impact of increased SNA resulting from microglial activation on hemodynamics and organ damage in sepsis associated with CKD.

**Methods and results:**

In protocol 1, male Sprague–Dawley rats underwent either nephrectomy (Nx) or sham surgery followed by cecal ligation and puncture (CLP) or sham surgery. After CLP, Nx-CLP rats exhibited decreased blood pressure, increased heart rate, elevated serum creatinine and bilirubin levels, and decreased platelet count compared to Nx-Sham rats. Heart rate variability analysis revealed an increased low to high frequency (LF/HF) ratio in Nx-CLP rats, indicating increased SNA. Nx-CLP rats also had higher creatinine and bilirubin levels and lower platelet counts than sham-CLP rats after CLP. In protocol 2, Nx-CLP rats were divided into two subgroups: one received minocycline, an inhibitor of microglial activation, while the other received artificial cerebrospinal fluid (CSF) intracerebroventricularly via an osmotic minipump. The minocycline-treated group (Nx-mino-CLP) showed attenuated hypotensive and increased heart rate responses compared to the CSF-treated group (Nx-CSF-CLP), and the LF/HF ratio was also decreased. Echocardiography showed larger left ventricular dimensions and inferior vena cava in the Nx-mino-CLP group. In addition, creatinine and bilirubin levels were lower and platelet counts were higher in the Nx-mino-CLP group compared to the Nx-CSF-CLP group.

**Conclusions:**

In septic rats with concomitant CKD, SNA was significantly enhanced and organ dysfunction was increased. It has been suggested that the mechanism of exacerbated organ dysfunction in these models may involve abnormal systemic hemodynamics, possibly triggered by activation of the central sympathetic nervous system through activation of microglia in the PVN.

**Supplementary Information:**

The online version contains supplementary material available at 10.1186/s40560-024-00742-2.

## Background

Sepsis is a global health crisis that affects approximately 49 million people worldwide and is thought to be responsible for 20% of all deaths annually [1]. Sepsis is now defined as life-threatening organ dysfunction resulting from an impaired host response to infection, highlighting the importance of organ dysfunction in its pathophysiology [2]. Infection triggers sepsis, with increased sympathetic nerve activity (SNA) in the early stages leading to excessive proinflammatory cytokines, metabolic problems, cardiac dysfunction, and organ dysfunction [3–6].

Microglia, the primary cells responsible for inflammation in the central nervous system [7, 8], have received considerable attention in the context of organ dysfunction associated with sepsis. Research has consistently reported that microglial activation is a major contributor to the pathogenesis of septic encephalopathy, also known as sepsis-associated brain dysfunction [9, 10]. In addition, microglia closely interact with neurons and play a pivotal role in several conditions, including neurodegenerative diseases, traumatic brain injury, and mental illness [11, 12]. In particular, they are actively involved in the regulation of SNA through the release of cytokines, chemokines and growth factors, making them critical contributors to the regulation of cardiovascular diseases, such as various forms of hypertension or heart failure [13–17].

Chronic kidney disease (CKD) comorbidities have been identified in epidemiologic studies as significant factors affecting the prognosis of sepsis, with individuals with CKD experiencing an increased incidence of sepsis-induced acute kidney injury and subsequent adverse outcomes [18, 19]. Similar to sepsis, sympathetic nervous system (SNS) activation plays a central role in the pathogenesis of CKD [20, 21]. Increased electrical activity in renal sympathetic afferents, driven by factors such as renal ischemia, elevated angiotensin II, renal oxidative stress and reduced nitric oxide production, activates cardiovascular control centers, primarily in the paraventricular nucleus of the hypothalamus (PVN), thereby systemically amplifying the SNS [22–24]. In our previous study, we used a 5/6 nephrectomy mouse model to reproduce CKD with hypertension. We found that this model exhibited a decrease in the inhibitory function of γ-aminobutyric acid in the PVN, resulting in increased central SNS activity [23]. In addition, we proposed that increased oxidative stress in the PVN may contribute to systemic SNA in the CKD model [25]. These findings suggest an important role for central nervous system dysregulation in driving increased systemic SNA in the pathogenesis of CKD. Taken together, excessive activation of the SNS is postulated as a potential contributing factor to the poor prognosis of sepsis coexisting with CKD. In particular, activation of the central SNS resulting from regulatory abnormalities in the hypothalamus may be intricately involved in this context. In the present study, we sought to elucidate the role of microglia in the PVN of animal models of sepsis with coexisting CKD and selectively administered the microglial activation inhibitor minocycline into the brain ventricles of CKD rats prior to induction of sepsis.

## Methods

### Animals and general procedures

Male Sprague–Dawley (SD) rats, 8 to 16 weeks old and weighing 360–480 g (Japan SLC, Inc., Hamamatsu, Japan) were used. Rats were fed standard chow and had free access to drinking water. The rats were individually housed in a room maintained at a constant temperature (22–23 °C) and humidity under a 12-h light/dark cycle (lights on between 09:00 and 21:00).

### Experimental protocol 1: enhanced sympathetic activation and end-organ damage in septic rats with comorbid chronic kidney disease

**Fig. 1:**
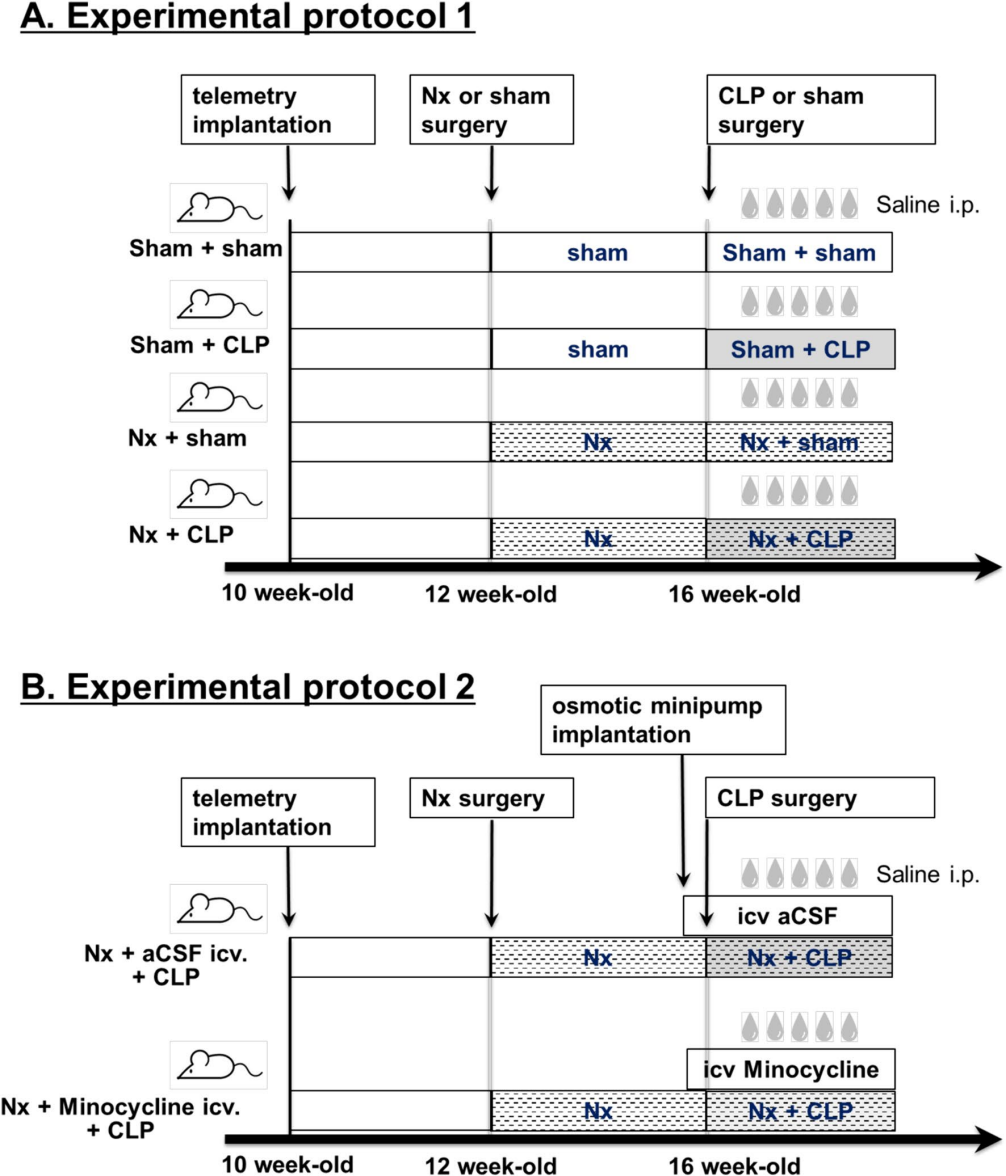
**A** Shows experimental protocol 1. Male Sprague–Dawley rats were implanted subcutaneously with electrocardiographic telemetry at 10 weeks of age and underwent either 5/6 nephrectomy (Nx) or sham surgery at 12 weeks of age. At 16 weeks of age, they underwent either cecal ligation and puncture (CLP) or sham surgery, resulting in four groups. Intraperitoneal (ip) saline was administered after CLP surgery and every 8 h thereafter. Several parameters were measured and compared between groups. **B** Shows protocol 2 for the CLP-induced experiment with intracerebroventricular (icv) administration of minocycline or artificial cerebrospinal fluid (aCSF) in Nx rats. Rats were implanted subcutaneously with electrocardiographic telemetry at 10 weeks of age, underwent Nx surgery at 12 weeks of age, and had osmotic minipumps implanted intracerebroventricularly two days before CLP surgery at 16 weeks of age. Several parameters were measured and compared between the two groups

Figure 1A shows the time series for each group in experimental protocol 1. Male SD rats were implanted with a telemetry system for electrocardiogram monitoring at 10 weeks of age. At 12 weeks of age, 5/6 nephrectomies (Nx), reproductive models of CKD, or sham surgeries (sham) were performed under 2.5% isoflurane anesthesia, with depth of anesthesia maintained by isoflurane inhalation (2.0–2.5%). The rats were observed for 4 weeks after surgery, and then the operated animals underwent either cecal ligation and puncture (CLP), replicated sepsis models, or sham surgery and were divided into 4 groups. The sham-operated group underwent the same surgical procedures except for CLP. Ketoprofen (5 mg/kg sc) was administered before each surgery.

We evaluated the following parameters over a two-day period following CLP surgery performed four weeks after either Nx or sham procedures. Food and water intake and urine output were measured for 24 h using a metabolic cage. Systolic blood pressure (BP) was measured using the tail-cuff method, and awake heart rate (HR) was assessed using electrocardiographic telemetry. Power spectral analysis of heart rate variability was performed to assess autonomic activity. Heart rate was continuously recorded with a wireless data acquisition system using PowerLab and analyzed using LabChart version 8 (ADInstruments, New Zealand). Heart rates were recorded at a sampling frequency of 1 kHz. Heart rate variability was assessed by analyzing the variation in pulse intervals. For frequency domain analysis, 5-min segment data were derived from the resting time in the light period. Low frequency (LF; 0.2–0.8 Hz) and high frequency (HF; 0.8–3.0 Hz) were calculated using LabChart. Sympathetic nerve activity was expressed as LF power in normalized units (LFnu), and parasympathetic nerve activity was expressed as HF power in normalized units (HFnu). The LF/HF ratio was calculated by dividing LF by HF to reflect the sympathovagal balance [26]. In addition, urinary norepinephrine excretion (uNE) was measured by 24-h urinalysis using a metabolic cage as an indicator of sympathetic activity. Cardiac function was evaluated by echocardiography by a single operator throughout the study. End organ damage was assessed by serum creatinine, total bilirubin, and platelet count.

Additional file 1: Supplemental Methods describes detailed methods in this section.

### Experimental protocol 2: association between excessive sympathetic hyperactivity caused by activation of brain microglia and organ damage in septic rats with comorbid chronic kidney disease

CLP (Nx-CLP) or sham (Nx-Sham) surgery was performed 4 weeks after Nx surgery to compare differences in microglial activation and neuronal activity in the hypothalamic PVN of the brain between the two groups. An ionized calcium-binding adaptor molecule 1 (Iba-1) immunohistochemical staining was performed to evaluate microglial activity in the PVN. Microglial activity in the PVN of the two groups was evaluated using ImageJ [27]. c-fos immunohistochemical staining was performed to evaluate neuronal activity (Additional file 1: Supplemental Methods for details).

To investigate the effects of microglial activity in the PVN on the enhancement of systemic SNA and organ damage in septic rats with CKD, we administered minocycline (Sigma–Aldrich, Saint Louis, USA), an inhibitor of microglial activity, intracerebroventricularly using an osmotic minipump prior to CLP (Nx-mino-CLP). As a control, an osmotic minipump was filled with artificial cerebrospinal fluid (aCSF) and administered before CLP (Nx-aCSF-CLP) (Additional file 1: Supplemental Methods for details). Figure 1B shows the time course of experimental protocol 2. The amount of minocycline (10 mg/ml) used was based on previous studies [13, 28]. In both Nx-mino-CLP and Nx-aCSF-CLP groups, microglial (Iba-1) and neuronal (c-fos) activity, body weight, water consumption, food intake, urine output, BP measured by tail cuff, awake HR by telemetry, and rectal temperature were measured on the first and second day of CLP. Frequency analysis using electrocardiographic telemetry and measurement of uNE were performed and compared to assess SNA. Various blood tests were performed to assess organ damage. Echocardiography was also performed to assess hemodynamic status. The results of these tests were compared between the two groups.

### Effects of intracerebroventricular administration of minocycline on sympathetic hyperactivity and organ damage induced by CLP in rats without chronic kidney disease

We investigated the effects of intracerebroventricular minocycline administration on SNS activation and organ damage in rats *without* comorbid CKD. Rats were implanted with electrocardiographic telemetry at 10 weeks of age, underwent sham surgery for 5/6 nephrectomy at 12 weeks of age, and had osmotic minipumps implanted at 16 weeks of age to initiate continuous intracerebroventricular administration of minocycline or aCSF. Subsequently, after performing CLP by the methods described above, we assessed SNS activation by heart rate variability analysis with electrocardiogram telemetry and assessment of uNE. We also monitored changes in BP over time and performed blood tests as indicators of organ damage.

### Statistical analyses

All results are presented as mean ± standard error of the mean. Data were analyzed by t-test or ANOVA followed by Bonferroni's correction for multiple comparisons. Statistical significance was considered at *p* < 0.05. Prism 9 (GraphPad Software Corp, San Diego, CA, USA) and EZR version 1.66 (Saitama Medical Center, Jichi Medical University, Saitama, Japan) were used for data analysis. In the present study, no a priori statistical sample size calculation was performed because of the difficulty in predicting the effect size. We used the number of animals generally used in the field for each experiment. We performed a post hoc power analysis to better interpret the results of experimental protocol 2.

## Results

### Validation of Nx-induced CKD characteristics

Table S1 summarizes the characteristics of the Nx rat group compared to the sham rat group 4 weeks after surgery. Nx resulted in a gradual increase in SBP that plateaued at 4 weeks. At this time point, Nx rats had significantly higher SBP and HR compared to age-matched controls. Plasma creatinine concentration and CCr showed significant increases and decreases, respectively, in Nx rats. Nx rats also had higher urinary albumin to creatinine ratio and uNE levels. Telemetry analysis revealed higher LFnu and LF/HF in Nx rats, with no significant difference in HFnu. Echocardiography showed smaller left ventricular end-systolic diameter (LVDs), greater LV wall thickness and higher percentage fractional shortening (%FS) in Nx rats. Inferior vena cava (IVC) diameter was smaller and collapsibility index tended to be higher in Nx rats. Nx rats had significantly reduced body weight, increased water intake and urine output, with no difference in food consumption.

### Effects of CLP on BP, HR, SNA, and end-organ damage

**Fig. 2:**
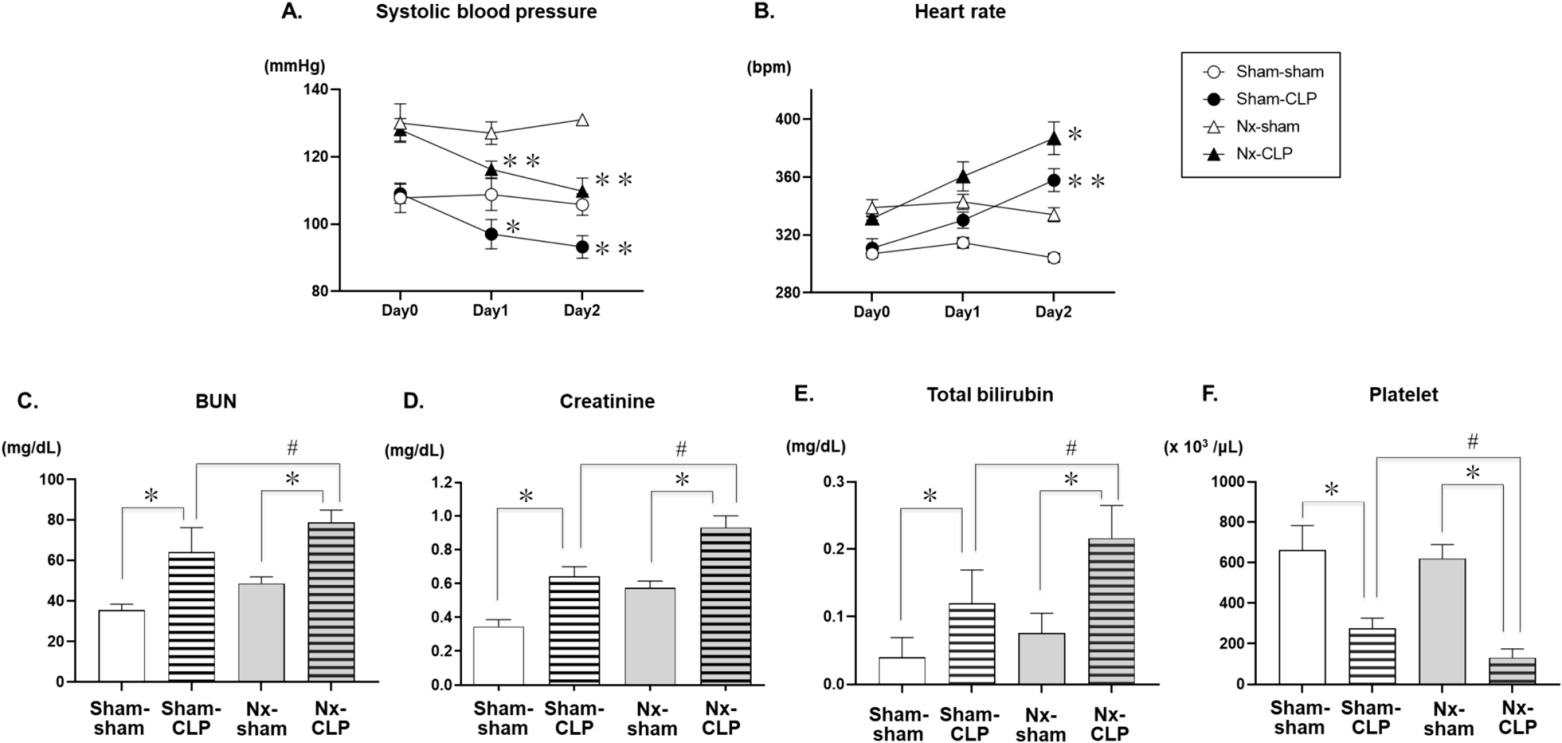
Time course of changes in systolic blood pressure (**A)** and heart rate (**B)** after cecal ligation and puncture (CLP) or sham surgery 4 weeks after 5/6 nephrectomy (Nx) or sham surgery. Values are mean ± SEM; *n* = 5 for each group. Two-way ANOVA with multiple comparisons, * p < 0.05, compared with each control (i.e., the comparison between the sham-CLP and sham-sham groups), **p < 0.01, compared with each control. Graphs **C**–**F** show the effects of CLP in Nx or control rats on blood test results. Data are expressed as mean ± SEM; *n* = 5 for each group. *P < 0.05, compared with sham operation of CLPs in each control group, and ^#^*P* < 0.05, compared with sham-CLP groups. *SEM* standard error of the mean, *ANOVA* analysis of variance

Four weeks after Nx or sham surgery, we divided the rats into four groups: Sham-Sham, Nx-Sham, Sham-CLP, and Nx-CLP. Blood pressure (SBP) was significantly lower on days 1 and 2 after CLP compared with sham surgery (Fig. 2A). Heart rate (HR) was significantly increased in the CLP group on days 1 and 2 after CLP (Fig. 2B), and body temperature was higher on days 1 and 2 (Additional file 2: Fig. S1). Two days after CLP or sham surgery, the CLP group had higher BUN, serum creatinine, total bilirubin, and lower platelet count. Nx-CLP rats had even higher serum creatinine, total bilirubin, and lower platelet counts than sham-CLP rats (Fig. 2C–F).

**Fig. 3:**
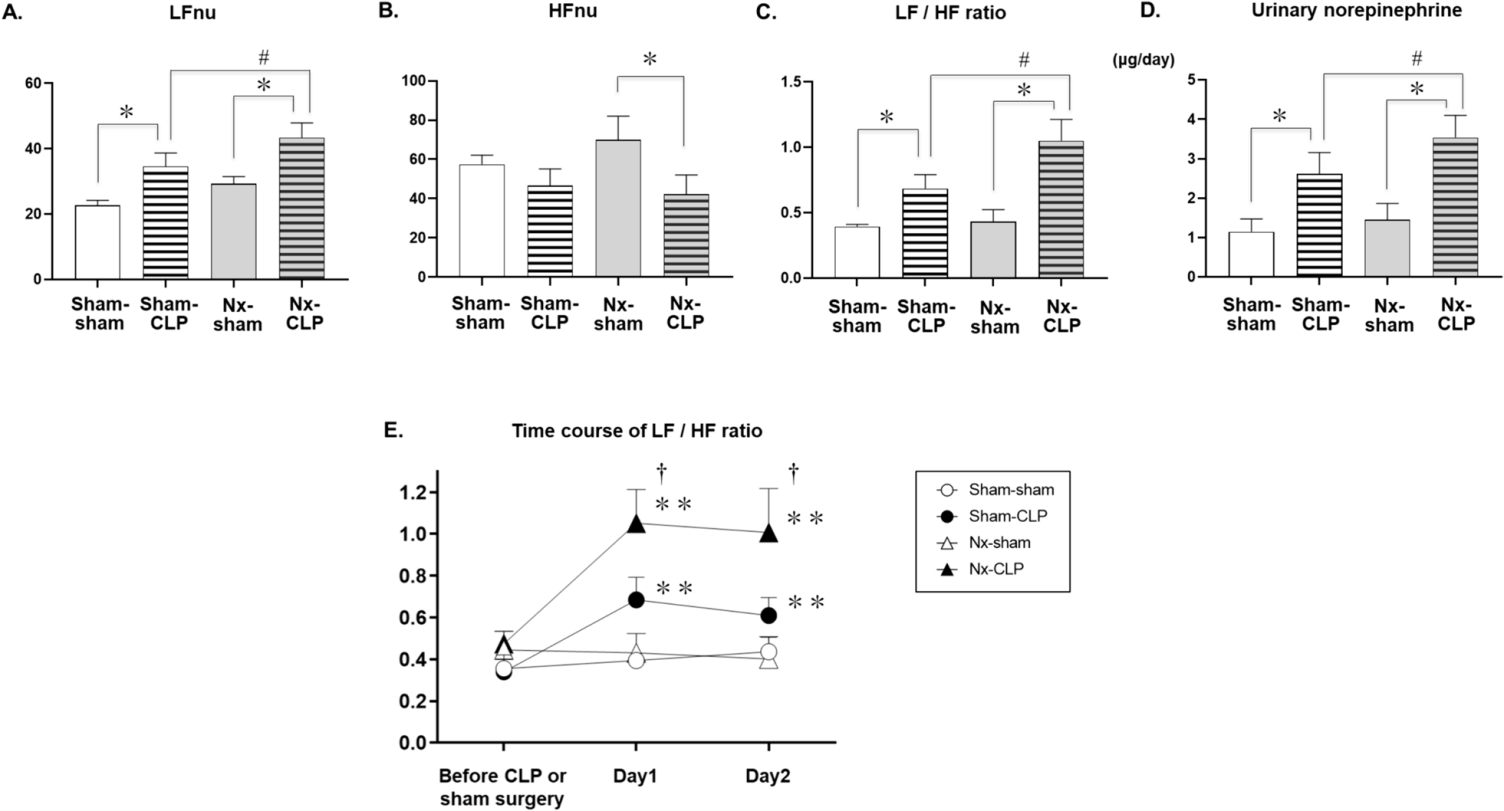
Graphs **A**–**C** show the effects of cecal ligation and puncture (CLP) in 5/6 nephrectomy (Nx) or control rats on the low-frequency component (LFnu), high-frequency component (HFnu), and LF/HF ratio obtained by analysis of heart rate data obtained by electrocardiographic telemetry. Graph **D** shows the effects of CLP in Nx or control rats on the urinary norepinephrine excretion level based on a 24-h urine collection. Data are expressed as mean ± SEM; sham-sham (*n* = 5), sham-CLP (*n* = 8), Nx-sham (*n* = 5), and Nx-CLP (*n* = 9) groups. *p < 0.01 compared with control groups, and ^#^*p* < 0.05 compared with sham-CLP groups. Graph **E** shows the time course (day1, day2) of the LF/HF ratio in Nx or sham-operated rats before and after the CLP procedure. Data are expressed as mean ± SEM. Two-way ANOVA with multiple comparisons, **p < 0.01, compared with each control, and ^†^*p* < 0.05, compared with sham-CLP. *SEM* standard error of the mean, *ANOVA* analysis of variance

Telemetry on day 1 after surgery showed higher LFnu in the CLP group compared to the corresponding sham group, and higher LFnu in Nx-CLP compared to sham-CLP (Fig. 3A). Nx-CLP had lower HFnu than Nx-sham (Fig. 3B). LF/HF was higher in sham-CLP and Nx-CLP compared to their respective shams on day 1 and 2, and higher in Nx-CLP than sham-CLP (Fig. 3C, E). uNE values were higher in the CLP group on day 1, and Nx-CLP had higher uNE than sham-CLP on day 1 (Fig. 3D).

Echocardiography (Additional file 2: Table S2) on day 2 showed smaller left ventricular dimensions (LVDd, LVDs) in sham-CLP and Nx-CLP compared to controls, with no significant differences between sham-CLP and Nx-CLP. Left ventricular wall thickness and %FS were not significantly different. IVC diameter was smaller in both sham-CLP and Nx-CLP compared to controls.

On day 2, body weight was similar in sham-CLP and sham-sham, while Nx-CLP showed a decreasing trend. Urine output was significantly lower in Nx-CLP compared to Nx-sham, and food intake was lower in both sham-CLP and Nx-CLP compared to controls. Water consumption decreased in Nx-CLP and showed a decreasing trend in sham-CLP (Additional file 2: Table S2).

### Activation of microglia and neuronal activity in the paraventricular nucleus of the hypothalamus in a rat model of CKD-complicated sepsis

**Fig. 4:**
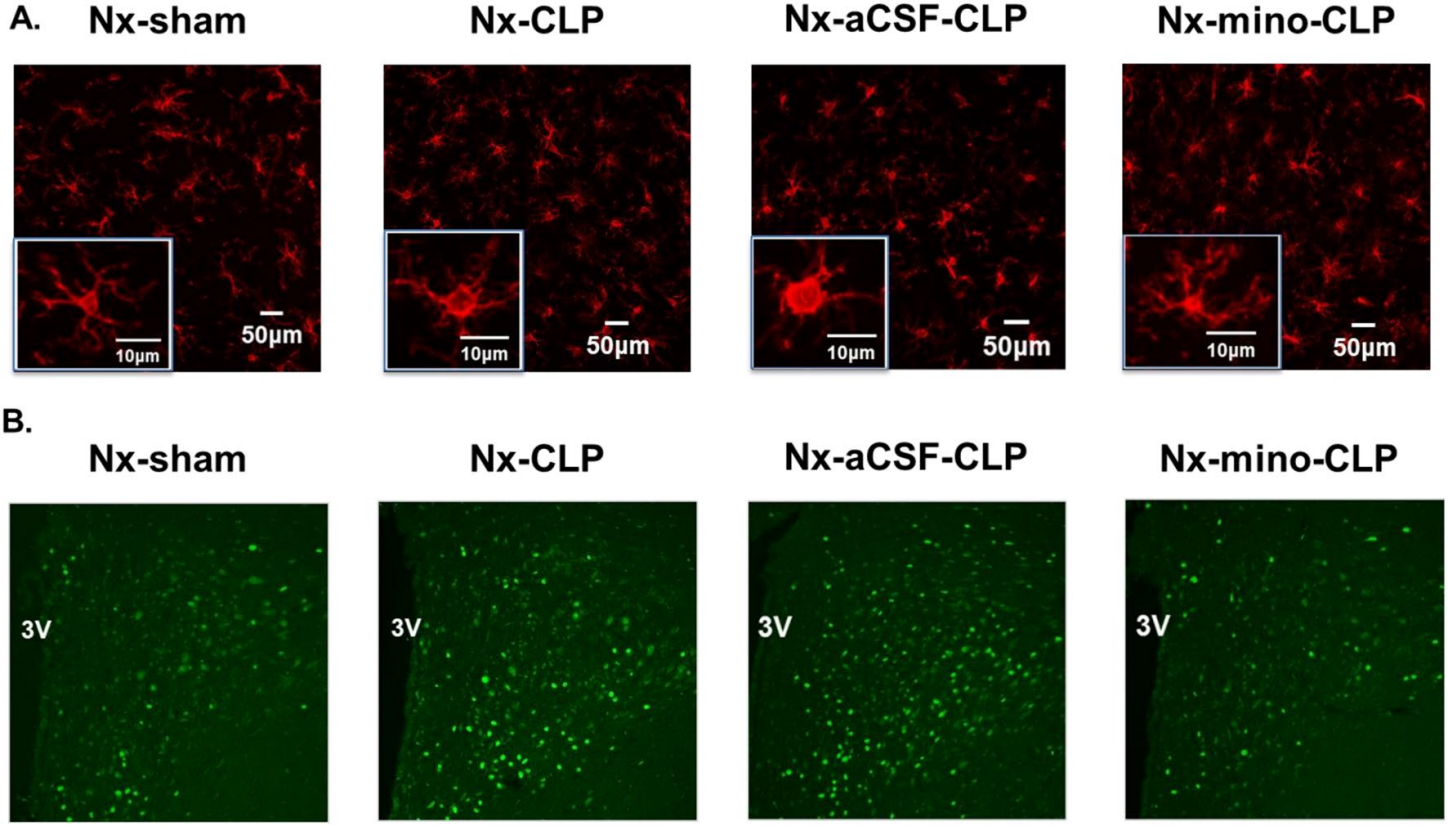
Effect of intracerebroventricular minocycline on microglia and neuronal activity in the paraventricular nucleus of the hypothalamus (PVN) of cecal ligation and puncture (CLP)-Induced 5/6 nephrectomy (Nx) rats. At the fourth week after Nx surgery, intracerebroventricular administration of minocycline (mino) or artificial cerebrospinal fluid (aCSF) was initiated, followed by CLP surgery (Nx-mino-CLP group and Nx-aCSF-CLP group). Graphs **A** show the results of immunohistochemical staining of macrophages with anti-Iba-1 antibody in the PVN in each group. Low and high magnification images are shown. Graphs **B** show the results of immunohistochemical staining of neurons in the PVN using anti-c-Fos antibody in each group. Iba-1 = ionized calcium-binding adaptor molecule 1, 3 V = third ventricle

Immunohistochemical staining for Iba-1 in the PVN of the brain (Localization of the PVN; Additional file 2: Fig. S2) showed that the perimeter and cell area of microglia were significantly attenuated and the circularity was significantly increased, indicating increased microglial activity, in Nx rats compared with the sham group on the second day after CLP (Fig. 4A and Additional file 2: Fig. S3). In Nx rats, c-fos activity in the hypothalamic paraventricular nucleus was significantly increased compared to the sham group on the second day after CLP surgery (Fig. 4B and Additional file 2: Fig. S3). These results suggest that microglial and neuronal activities in the hypothalamic paraventricular nucleus are activated by CLP in Nx rat.

### Effects of intracerebroventricular administration of microglial inhibitor on BP, HR, SNA, and end-organ damage in a rat model of CKD complicated sepsis

At 4 weeks after 5/6 nephrectomy, Nx rats were implanted with osmotic minipumps filled with either minocycline or aCSF and divided into two groups for subsequent CLP under continuous intracerebroventricular administration (Nx-mino-CLP; *n* = 5, Nx-aCSF-CLP; *n* = 5). Immunohistochemical staining for Iba-1 and c-fos in the PVN of the brain revealed that microglial activity (Fig. 4A and Additional file 2: Fig. S4) and neuronal activation (Fig. 4B and Additional file 2: Fig. S4) at the same site were significantly inhibited in the Nx-mino-CLP group compared to the Nx-aCSF-CLP group.

**Fig. 5:**
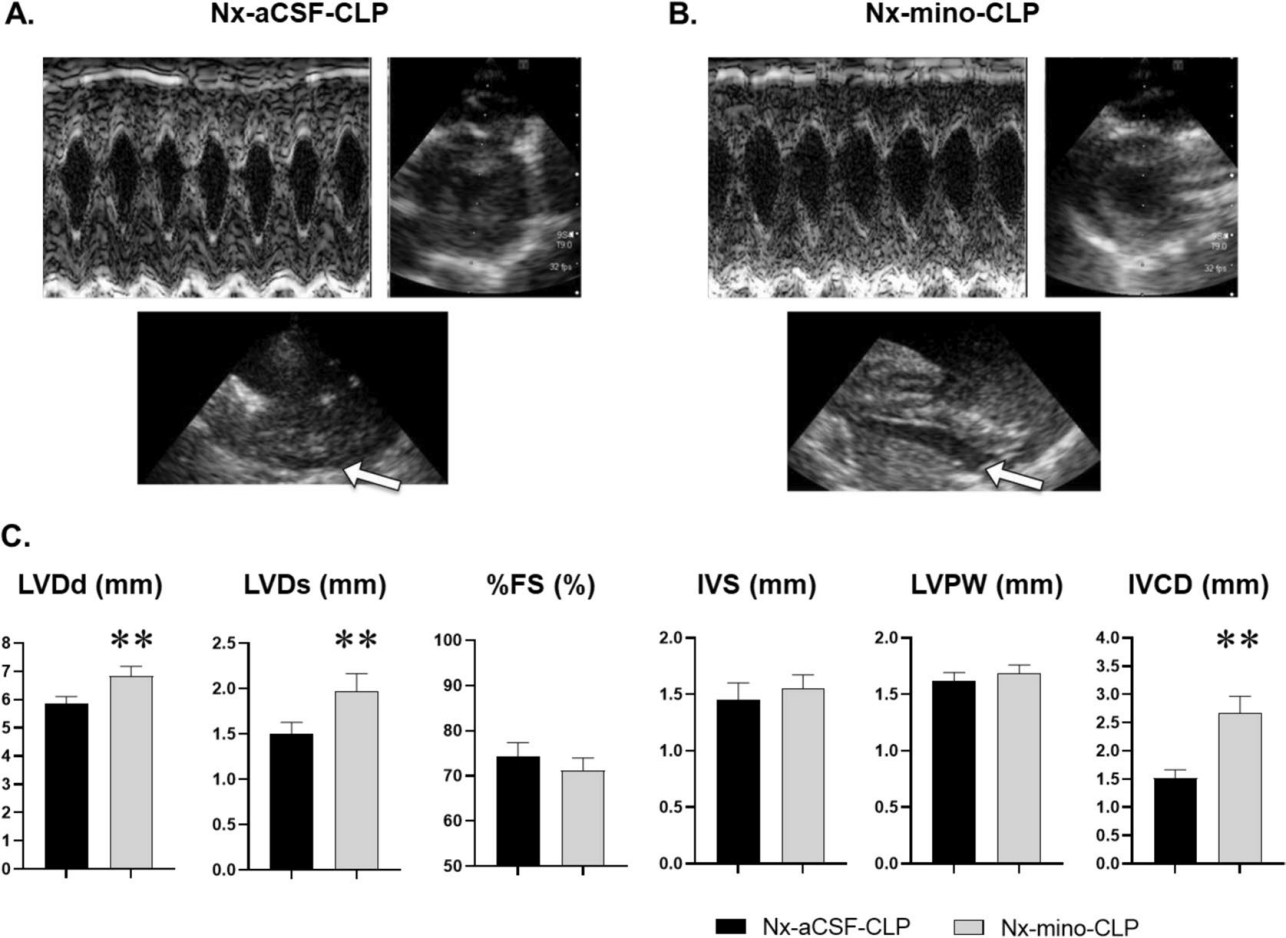
Effect of intracerebroventricular minocycline on echocardiographic changes in cecal ligation and puncture (CLP)-Induced 5/6 nephrectomy (Nx) rats. At the fourth week after Nx surgery, intracerebroventricular administration of minocycline (mino) or artificial cerebrospinal fluid (aCSF) was initiated, followed by CLP surgery (Nx-mino-CLP group and Nx-aCSF-CLP group). Graphs **A** and **B** show the echocardiographic imaging results on the second day after CLP surgery in the Nx-aCSF-CLP and Nx-mino-CLP groups, respectively. In graphs **A** and **B**, the upper left panel shows M-mode images at the level of the left ventricular papillary muscles, the upper right panel shows short-axis images at end-diastole of the left ventricle, and the lower panel shows IVC images. The arrow in the diagram points to the IVC. Graph **C** shows the echocardiographic measurements for both groups. Values are mean ± SEM; n = 6 for each group. **p < 0.01, in comparison with Nx-aCSF-CLP rats. *LVDd* LV end-diastolic diameter, *LVDs* LV end-systolic diameter, *%FS* percent fractional shortening, *IVS* interventricular septum thickness, *LVPW *left ventricular posterior wall thickness, *IVCD* inferior vena cava diameter, and *SEM* standard error of the mean

After CLP, Nx-mino-CLP had a significantly attenuated decrease in BP on day 2 compared to Nx-aCSF-CLP. The increase in HR after CLP was significantly suppressed on days 1 and 2 in Nx-mino-CLP compared to Nx-aCSF-CLP (Table 1 and Additional file 2: Fig. S5). On day 2, the increase in rectal temperature was significantly suppressed in Nx-mino-CLP (Additional file 2: Fig. S5). Blood tests showed significantly lower BUN, creatinine and total bilirubin in Nx-mino-CLP and higher platelet count in Nx-mino-CLP compared to Nx-aCSF-CLP (Table 1). Urinary albumin excretion was not different between the two groups (0.26 ± 0.02 mg/g ∙ creatinine vs. 0.29 ± 0.03 mg/g ∙ creatinine, *n* = 5 for each, *p* > 0.05). These results suggest that organ damage after CLP was ameliorated in the Nx-mino-CLP group compared with the Nx-aCSF-CLP group. Telemetry on postoperative day 1 showed significantly lower LFnu and higher HFnu in Nx-mino-CLP (Table 1). On days 1 and 2 after CLP, the LF/HF ratio was significantly lower in Nx-mino-CLP (Additional file 2: Fig. S6). uNE levels were significantly lower in Nx-mino-CLP (Table 1). These results indicate suppressed SNS and enhanced parasympathetic activity in Nx-mino-CLP from the early post-CLP period compared to Nx-aCSF-CLP. Echocardiography revealed significantly larger LVDd, LVDs, and IVC diameter in Nx-mino-CLP vs. Nx-aCSF-CLP (Fig. 5). Interventricular septum thickness, left ventricular posterior wall thickness, and %FS were similar between groups. These findings suggest a better preserved blood volume in Nx-mino-CLP after CLP. After CLP, Nx-mino-CLP showed a trend toward increased urine volume and water intake, with no differences in body weight and food intake compared to Nx-aCSF-CLP (Table 1). The results of the post hoc statistical power analysis of the effects of intracerebroventricular administration of a minocycline in a rat model of CKD complicated sepsis (a sample size of five rats per group) are presented in Supplementary Table 4 in Additional file 2.

**Table 1:** Effects of intracerebroventricular administration of microglial inhibitor in a rat model of CKD complicated sepsis

Variables	Nx-aCSF-CLP rats (*n* = 5)	Nx-mino-CLP rats (*n* = 5)	*p*
Hemodynamics			
Systolic blood pressure (mm Hg)	111 ± 2	120 ± 2	0.02
Heart rate (per minute)	397 ± 8	362 ± 4	0.008
Blood test			
Blood urea nitrogen (mg/dL)	79 ± 3	61 ± 5	0.02
Plasma creatinine concentration (mg/dL)	0.95 ± 0.09	0.72 ± 0.03	0.03
Total bilirubin (mg/dL)	0.20 ± 0.02	0.13 ± 0.01	0.02
Platelet count (× 10^3^ /μL)	198 ± 25	309 ± 33	0.03
Heart rate variability analysis			
LFnu	43.6 ± 1.7	35.8 ± 2.7	0.04
HFnu	41.5 ± 1.5	55.0 ± 3.4	0.007
LF/HF ratio	1.05 ± 0.08	0.76 ± 0.03	0.008
Urinary norepinephrine (µg/day)	3.7 ± 0.2	2.7 ± 0.2	0.07
Physiological parameters			
Body weight (g)	435 ± 4	441 ± 5	0.55
Urine volume (ml)	12 ± 2	17 ± 1	0.064
Water intake (ml)	28 ± 2	35 ± 2	0.095
Food intake (g)	4 ± 1	5 ± 1	0.83

*CLP* cecal ligation and puncture, *Nx* 5/6 nephrectomy, *aCSF* artificial cerebrospinal fluid, and mino; minocycline, *LFnu* low-frequency power in normalized units, *HFnu* high-frequency power in normalized units. Values are mean ± SEM

### Effects of intracerebroventricular administration of a microglial inhibitor on BP, HR, SNA, and end-organ damage in a rat model of non-CKD complicated sepsis

Four weeks after sham surgery for 5/6 nephrectomy, sham rats were implanted with osmotic minipumps filled with either minocycline or aCSF and divided into two groups for subsequent CLP under continuous intracerebroventricular administration (sham-mino-CLP; *n* = 5, sham-aCSF-CLP; *n* = 5). Both groups showed a decrease in BP and an increase in HR after CLP, but no significant difference was observed between the two groups. Both groups showed an increase in rectal temperature after CLP, but no significant difference was observed (Additional file 2: Fig. S7). Blood tests showed that BUN, creatinine, total bilirubin levels, and platelet count were not statistically different between the two groups (Additional file 2: Fig. S8). Heart rate variability analysis using telemetry on postoperative day 1 revealed that LFnu, HFnu, and LF/HF ratio were not statistically different between sham-mino-CLP and sham-aCSF-CLP groups. In addition, uNE levels were not significantly different between the two groups (Additional file 2: Fig. S9).

## Discussion

As a novel finding in the present study, we observed an enhancement of SNS activity and exacerbation of organ damage in septic rats coexisting with CKD compared to septic rats without CKD. Septic rats with coexisting CKD showed activation of microglia and neurons in the PVN of the hypothalamus. Furthermore, when a microglial inhibitor was selectively administered to the brain ventricles of rats exhibiting the pathophysiology of CKD, followed by induction of sepsis, the increase in SNS activity and the degree of organ damage were attenuated. These results demonstrate the significant role of central SNS activation, originating from microglial activation in the brain, in the pathogenesis of sepsis in rats coexisting with CKD.

### CKD rats undergo CLP surgery

In this study, male SD rats underwent 5/6 nephrectomy to induce the pathological features of CKD. SD rats exhibited characteristic features of CKD, including a decrease in creatinine clearance along with hypertension and increased urinary albumin excretion. Furthermore, as previously reported, uNE levels as a marker of SNA were significantly elevated in Nx rats [23, 29, 30]. CKD is characterized by sympathetic overactivity not only in experimental animals but also in patients [21]. In CKD patients, cardiovascular events are strongly associated with increased sympathetic activity [31–33]. Left ventricular enlargement and wall thickening associated with hypertension were confirmed by echocardiography in Nx rats. As previously reported, this model showed increased urine volume and water intake [34, 35]. In essence, the CKD model used in this study exhibited characteristics similar to those of human CKD patients. In addition, we performed CLP surgery to induce sepsis. In this study, as presented in the results section, we observed hemodynamic changes and organ damage after CLP that were indicative of a septic state. These findings were consistent with previous research [36, 37].

### Brain microglia inhibition attenuates SNA and organ dysfunction

Selective inhibition of microglial activity in the cerebral ventricles before CLP in Nx rats demonstrated suppression of SNA and organ dysfunction after CLP. The role of brain microglia in sepsis has been reported to be associated with sepsis-associated encephalopathy (SAE). A study by Michels et al. demonstrated the involvement of microglia in brain inflammation and oxidative stress during SAE [38]. Furthermore, preoperative intracerebroventricular administration of minocycline inhibited oxidative stress and increased inflammatory markers in the hippocampus after CLP, leading to improved cognitive function [38]. However, research on SAE has mainly focused on investigating the effects of activated microglia on brain-specific functions such as cognitive function, and their effects on organs other than the brain remain unclear. In contrast, in pathologies other than sepsis, such as hypertension [13–16], myocardial infarction, and heart failure [17], microglial activation in the brain has long been reported to contribute significantly to systemic sympathetic activation, leading to organ dysfunction. Shi P et al. [13] demonstrated that intracerebroventricular minocycline administration in hypertensive rats suppressed cardiac hypertrophy, and plasma norepinephrine, while Yu XJ et al. [15] showed that sustained minocycline in the PVN region of the brain reduced BP response and suppressed microglial and sympathetic nerve activities in rats on a high-salt diet. These studies consistently indicate that brain microglial activation is deeply involved in various pathological processes through the enhancement of systemic SNS activity in chronic diseases. Furthermore, in the present study, we demonstrated the effects of intracerebroventricular minocycline administration on systemic organ damage during the acute phase of sepsis. In the setting of the acute phase of sepsis, previous studies have also reported the organ-protective effects of systemic administration of beta-blockers, which inhibit sympathetic activity, or alpha-2 agonists, which suppress central sympathetic activation [4, 39]. These studies suggest that suppression of increased sympathetic activity during the acute phase of sepsis may inhibit the progression of organ damage through its effects on hemodynamics, the immune system, or the coagulation system. In our experimental model, it is plausible that suppression of excessive sympathetic activation by intracerebroventricular minocycline administration during the acute phase of sepsis with coexisting CKD may result in such a multiple effect leading to attenuation of organ damage.

### Rebalancing autonomic nervous system toward parasympathetic activation

Another interesting finding in the present study was that in septic rats with concomitant CKD, the parasympathetic nervous system component assessed by heart rate variability analysis was significantly decreased compared with the sham surgery group. However, when CLP was performed after prior intracerebroventricular administration of minocycline, the decrease in parasympathetic component was not observed compared with the control group pretreated with cerebrospinal fluid. These results suggest that suppressing microglial activity in the brain in the CKD model may correct the imbalance in the autonomic nervous system favoring sympathetic dominance induced by CLP. Several previous studies have demonstrated the prognostic benefits of parasympathetic activation in sepsis. Galantamine, a cholinesterase inhibitor, administered before lipopolysaccharide (LPS) injection, reduced lung injury assessed after 12 h of LPS injection and increased survival in mice through central acetylcholinesterase inhibition and activation of the vagal anti-inflammatory pathway [40, 41]. Classical cholinesterase inhibitors, physostigmine and neostigmine, also increased survival in endotoxemia or sepsis [42], with neostigmine being able to enter the brain due to the disruption of the blood–brain barrier in sepsis [43]. In addition, previous experimental studies have shown that cholinergic or vagal nerve stimulation inhibits cytokine production and activity and improves disease endpoints such as reduced norepinephrine requirements or end-organ damage in a number of experimental rodent models of sepsis [44–46]. Previous reports have shown that peripheral vagus nerve stimulation before and after administration of a lethal dose of LPS to rats suppressed the hypotensive response observed one hour after LPS administration, as well as the production of tumor necrosis factor (TNF) in serum and liver tissue [44]. Furthermore, in a porcine model of peritonitis-induced sepsis, vagus nerve stimulation initiated 6 h after induction of peritonitis was associated with a significant reduction in multiple organ dysfunction and a decreased need for vasopressors and fluids at 24 h [46]. These studies show that activation of the parasympathetic nervous system from the early stages of sepsis has a suppressive effect on the progression of organ damage in sepsis, and our current study results may also support this.

### Possible mechanisms

The specific mechanisms by which inhibition of central SNS activity by suppressing microglia prevents the progression of organ damage in sepsis are not fully understood. In sepsis, increased sympathetic activity leads to increased norepinephrine release, which downregulates alpha receptors on arteries and decreases vascular reactivity to released norepinephrine, requiring increased vasopressors [47, 48]. Clonidine or dexmedetomidine, both alpha 2 agonists, have a potent central sympathetic inhibitory effect, potentially restoring norepinephrine responsiveness in the peripheral vasculature [47, 48], and central sympathetic inhibition by the current approach may have had a similar effect. The echocardiographic results in our study showed a significant increase in left ventricular internal diameter and IVC diameter in the sepsis-induced model after minocycline preadministration, suggesting that vascular volume may be preserved by restoring vascular responsiveness to endogenous norepinephrine compared with the control CSF-treated group. These effects suggest a possible involvement in the amelioration of hemodynamic abnormalities and organ damage induced by CLP after minocycline intracerebroventricular preadministration in the CKD model. Further studies are needed to clarify this issue.

## Limitations

The present study has several limitations. First, we did not directly assess neural activity as a measure of SNS activity. After CLP surgery, the increased inflammatory state associated with infection made the assessment of highly invasive direct SNS activity challenging. However, in this study we performed an analysis of autonomic heart rate variability during wakefulness using telemetry in addition to urinary norepinephrine excretion as an indicator of sympathetic nervous system activity (a methodology established in previous research). These results consistently support the findings of SNS activity in the present study and lend robustness to our findings. Second, we examined the specific effects of CLP only on the PVN. Cardiovascular regulation is controlled by several critical nuclei in the brain, including the subfornical organ, nucleus tractus solitarii, and rostral ventrolateral medulla, which interact with each other. The influence of CLP on microglia in the brain may not be limited to the PVN. However, the studies have demonstrated the significant role of microglia in this region in various pathological processes [13–16]. Given the well-established fact that SNS activity is strongly regulated by PVN activation [23, 25, 49, 50], this study focused on the impact of microglial activation in the PVN on systemic SNS activity and its contribution to the pathogenesis of sepsis. Third, the present study did not evaluate the effects of the CLP procedure on the central nervous system and respiratory status, the usual clinical indicator of organ dysfunction in sepsis, in CKD rats. The evaluation focused on organ dysfunction using indicators such as blood pressure, echocardiography, serum creatinine levels, serum total bilirubin levels, and platelet counts. Sepsis-associated encephalopathy, a common complication, was recognized [51] and microglial activation in the central nervous system was considered [9, 10]. The invasive nature of the CLP procedure may have affected consciousness and respiratory status, suggesting the need for further research. Fourth, in this study, we investigated the relationship between microglial activation in the PVN and sympathetic nervous system activity and organ damage in sepsis coexisting with CKD by inhibiting microglial activities in the PVN; however, to rigorously investigate this relationship, it is necessary to intentionally activate microglia in the PVN in this model and evaluate the resulting organ damage. In our CKD-complicated CLP model, the procedure itself leads to microglial activation in the brain, making further activation and evaluation challenging. Further research is needed to clarify this point. Lastly, the detailed mechanisms by which microglial activation in the brain stimulates neuronal activation were not elucidated in this study. We observed microglial activation in the brains of CKD coexisting rats after CLP surgery. Selective inhibition of microglial activation also attenuated neuronal activity as indicated by c-fos expression, raising the possibility that CLP-induced microglial activation leads to subsequent neuronal activation through some form of interaction. Previous study has reported that systemic administration of LPS activates microglia in the PVN, thereby promoting the production of proinflammatory cytokines within the PVN. These proinflammatory cytokines have been reported to enhance the excitation of presympathetic parvocellular neurons in the PVN, resulting in excessive SNS activity [52]. In the septic rats with coexisting CKD used in this study, it remains to be determined how microglia can activate neurons through specific signals and molecular pathways, making this a subject for future research. Further studies are needed to clarify these important issues.

## Conclusion

The results of the present study demonstrate a compelling association between sepsis and increased organ damage in rats with CKD compared to those without CKD. This increased damage could be attributed, at least in part, to abnormal hemodynamics driven by excessive sympathetic nervous system activity induced by microglial activation within the PVN of the brain in septic rats with comorbid CKD. In this disease model, correcting the imbalance between the sympathetic and parasympathetic nervous systems attenuated organ damage, suggesting that these autonomic nervous system abnormalities are deeply involved in the pathogenesis of sepsis with CKD. The specific roles of the sympathetic and parasympathetic nervous systems in this disease model are not fully understood and remain important topics for future research.

### Supplementary Information

Additional file 1

Additional file 2. 

## Data Availability

The data supporting the conclusions of this article are included within the article and its additional files.

## Abbreviations

SNA: Sympathetic nerve activity
CKD: Chronic kidney disease
SNS: Sympathetic nervous system
PVN: Paraventricular nucleus of the hypothalamus
SD rats: Sprague–Dawley rats
Nx: Nephrectomy
CLP: Cecal ligation and puncture
CCr: Creatinine clearance
LF: Low frequency
HF: High frequency
uNE: Urinary norepinephrine
aCSF: Artificial cerebrospinal fluid
BP: Blood pressure
HR: Heart rate
LVDs: Left ventricular end-systolic diameter
%FS: Percentage fractional shortening
LVDd: Left ventricular end-diastolic diameter
IVC: Inferior vena cava
LPS: Lipopolysaccharide

## References

[1] Rudd KE, Johnson SC, Agesa KM, Shackelford KA, Tsoi D, Kievlan DR, et al. Global, regional, and national sepsis incidence and mortality, 1990–2017: analysis for the Global Burden of Disease Study. Lancet. 2020;395(10219):200–11. 31954465 10.1016/S0140-6736(19)32989-7 PMC6970225

[2] Singer M, Deutschman CS, Seymour CW, Shankar-Hari M, Annane D, Bauer M, et al. The third international consensus definitions for sepsis and septic shock (Sepsis-3). JAMA. 2016;315(8):801–10. 26903338 10.1001/jama.2016.0287 PMC4968574

[3] Palsson J, Ricksten SE, Delle M, Lundin S. Changes in renal sympathetic nerve activity during experimental septic and endotoxin shock in conscious rats. Circ Shock. 1988;24(2):133–41. 3286033

[4] Petitjeans F, Geloen A, Pichot C, Leroy S, Ghignone M, Quintin L. Is the sympathetic system detrimental in the setting of septic shock, with antihypertensive agents as a counterintuitive approach? A clinical proposition. J Clin Med. 2021;10(19):4569. 34640590 10.3390/jcm10194569 PMC8509206

[5] Kawaguchi S, Okada M. Cardiac metabolism in sepsis. Metabolites. 2021;11(12):846. 34940604 10.3390/metabo11120846 PMC8707959

[6] Lin H, Wang W, Lee M, Meng Q, Ren H. Current status of septic cardiomyopathy: basic science and clinical progress. Front Pharmacol. 2020;11:210. 32194424 10.3389/fphar.2020.00210 PMC7062914

[7] Michels M, Danielski LG, Dal-Pizzol F, Petronilho F. Neuroinflammation: microglial activation during sepsis. Curr Neurovasc Res. 2014;11(3):262–70. 24845857 10.2174/1567202611666140520122744

[8] Hoogland IC, Houbolt C, van Westerloo DJ, van Gool WA, van de Beek D. Systemic inflammation and microglial activation: systematic review of animal experiments. J Neuroinflammation. 2015;12:114. 26048578 10.1186/s12974-015-0332-6 PMC4470063

[9] Yan X, Yang K, Xiao Q, Hou R, Pan X, Zhu X. Central role of microglia in sepsis-associated encephalopathy: From mechanism to therapy. Front Immunol. 2022;13: 929316. 35958583 10.3389/fimmu.2022.929316 PMC9361477

[10] Hu J, Xie S, Zhang H, Wang X, Meng B, Zhang L. Microglial activation: key players in sepsis-associated encephalopathy. Brain Sci. 2023;13(10):1453. 37891821 10.3390/brainsci13101453 PMC10605398

[11] Wolf SA, Boddeke HW, Kettenmann H. Microglia in physiology and disease. Annu Rev Physiol. 2017;79:619–43. 27959620 10.1146/annurev-physiol-022516-034406

[12] Strogulski NR, Portela LV, Polster BM, Loane DJ. Fundamental neurochemistry review: microglial immunometabolism in traumatic brain injury. J Neurochem. 2023;167(2):129–53. 37759406 10.1111/jnc.15959 PMC10655864

[13] Shi P, Diez-Freire C, Jun JY, Qi Y, Katovich MJ, Li Q, et al. Brain microglial cytokines in neurogenic hypertension. Hypertension. 2010;56(2):297–303. 20547972 10.1161/HYPERTENSIONAHA.110.150409 PMC2929640

[14] Mi Y, Wu Q, Yuan W, Chen F, Du D. Role of microglia M1/M2 polarisation in the paraventricular nucleus: New insight into the development of stress-induced hypertension in rats. Auton Neurosci. 2018;213:71–80. 30005742 10.1016/j.autneu.2018.06.003

[15] Yu XJ, Liu XJ, Guo J, Su YK, Zhang N, Qi J, et al. Blockade of microglial activation in hypothalamic paraventricular nucleus improves high salt-induced hypertension. Am J Hypertens. 2022;35(9):820–7. 35439285 10.1093/ajh/hpac052

[16] Cheng L, Correia MLG. More evidence links microglia and neuroinflammation with hypertension. Am J Hypertens. 2022;35(9):787–9. 35815792 10.1093/ajh/hpac081

[17] Diaz HS, Toledo C, Andrade DC, Marcus NJ, Del Rio R. Neuroinflammation in heart failure: new insights for an old disease. J Physiol. 2020;598(1):33–59. 31671478 10.1113/JP278864

[18] Leelahavanichkul A, Huang Y, Hu X, Zhou H, Tsuji T, Chen R, et al. Chronic kidney disease worsens sepsis and sepsis-induced acute kidney injury by releasing High Mobility Group Box Protein-1. Kidney Int. 2011;80(11):1198–211. 21832986 10.1038/ki.2011.261 PMC3491658

[19] Doi K. Role of kidney injury in sepsis. J Intensive Care. 2016;4:17. 27011788 10.1186/s40560-016-0146-3 PMC4804517

[20] Salman IM. Cardiovascular autonomic dysfunction in chronic kidney disease: a comprehensive review. Curr Hypertens Rep. 2015;17(8):59. 26071764 10.1007/s11906-015-0571-z

[21] Grassi G, Biffi A, Seravalle G, Bertoli S, Airoldi F, Corrao G, et al. Sympathetic nerve traffic overactivity in chronic kidney disease: a systematic review and meta-analysis. J Hypertens. 2021;39(3):408–16. 33031182 10.1097/HJH.0000000000002661

[22] DiBona GF, Kopp UC. Neural control of renal function. Physiol Rev. 1997;77(1):75–197. 9016301 10.1152/physrev.1997.77.1.75

[23] Nishihara M, Takesue K, Hirooka Y. Renal denervation enhances GABA-ergic input into the PVN leading to blood pressure lowering in chronic kidney disease. Auton Neurosci. 2017;204:88–97. 27729205 10.1016/j.autneu.2016.09.018

[24] Sata Y, Head GA, Denton K, May CN, Schlaich MP. Role of the sympathetic nervous system and its modulation in renal hypertension. Front Med (Lausanne). 2018;5:82. 29651418 10.3389/fmed.2018.00082 PMC5884873

[25] Nishihara M, Takesue K, Hirooka Y. Olmesartan combined with renal denervation reduces blood pressure in association with sympatho-inhibitory and aldosterone-reducing effects in hypertensive mice with chronic kidney disease. Clin Exp Hypertens. 2019;41(3):211–9. 29694249 10.1080/10641963.2018.1465075

[26] Kuwahara M, Yayou K, Ishii K, Hashimoto S, Tsubone H, Sugano S. Power spectral analysis of heart rate variability as a new method for assessing autonomic activity in the rat. J Electrocardiol. 1994;27(4):333–7. 7815012 10.1016/S0022-0736(05)80272-9

[27] Young K, Morrison H. Quantifying microglia morphology from photomicrographs of immunohistochemistry prepared tissue using imageJ. J Vis Exp. 2018;136:57648. 10.3791/57648 PMC6103256 29939190

[28] Xue B, Thunhorst RL, Yu Y, Guo F, Beltz TG, Felder RB, et al. Central renin-angiotensin system activation and inflammation induced by high-fat diet sensitize angiotensin II-elicited hypertension. Hypertension. 2016;67(1):163–70. 26573717 10.1161/HYPERTENSIONAHA.115.06263 PMC4834194

[29] Yuhara M, Ikeda T, Toya Y, Sakurai J, Gomi T, Ikeda T. Participation of the sympathetic nervous system in hypertension in rats with subtotal renal ablation. J Hypertens. 1989;7(6):443–6. 2778311 10.1097/00004872-198906000-00002

[30] Dugaich AP, Oliveira-Sales EB, Abreu NP, Boim MA, Bergamaschi CT, Campos RR. Role of the rostral ventrolateral medulla in the arterial hypertension in chronic renal failure. Int J Hypertens. 2011;2010: 219358. 21253520 10.4061/2010/219358 PMC3022169

[31] Zoccali C, Mallamaci F, Parlongo S, Cutrupi S, Benedetto FA, Tripepi G, et al. Plasma norepinephrine predicts survival and incident cardiovascular events in patients with end-stage renal disease. Circulation. 2002;105(11):1354–9. 11901048 10.1161/hc1102.105261

[32] Schlaich MP, Socratous F, Hennebry S, Eikelis N, Lambert EA, Straznicky N, et al. Sympathetic activation in chronic renal failure. J Am Soc Nephrol. 2009;20(5):933–9. 18799718 10.1681/ASN.2008040402

[33] Kaur J, Young BE, Fadel PJ. Sympathetic overactivity in chronic kidney disease: consequences and mechanisms. Int J Mol Sci. 2017;18(8):1682. 28767097 10.3390/ijms18081682 PMC5578072

[34] Gava AL, Freitas FP, Balarini CM, Vasquez EC, Meyrelles SS. Effects of 5/6 nephrectomy on renal function and blood pressure in mice. Int J Physiol Pathophysiol Pharmacol. 2012;4(3):167–73. 23071874 PMC3466491

[35] Chang D, Xu TT, Zhang SJ, Cai Y, Min SD, Zhao Z, et al. Telmisartan ameliorates cardiac fibrosis and diastolic function in cardiorenal heart failure with preserved ejection fraction. Exp Biol Med (Maywood). 2021;246(23):2511–21. 34342551 10.1177/15353702211035058 PMC8649930

[36] Hollenberg SM, Dumasius A, Easington C, Colilla SA, Neumann A, Parrillo JE. Characterization of a hyperdynamic murine model of resuscitated sepsis using echocardiography. Am J Respir Crit Care Med. 2001;164(5):891–5. 11549551 10.1164/ajrccm.164.5.2010073

[37] Ren C, Li XH, Wu Y, Dong N, Tong YL, Yao YM. Inhibition of cerebral high-mobility group box 1 protein attenuates multiple organ damage and improves T cell-mediated immunity in septic rats. Mediators Inflamm. 2019;2019:6197084. 30881224 10.1155/2019/6197084 PMC6387733

[38] Michels M, Vieira AS, Vuolo F, Zapelini HG, Mendonca B, Mina F, et al. The role of microglia activation in the development of sepsis-induced long-term cognitive impairment. Brain Behav Immun. 2015;43:54–9. 25019583 10.1016/j.bbi.2014.07.002

[39] Suzuki T, Suzuki Y, Okuda J, Kurazumi T, Suhara T, Ueda T, et al. Sepsis-induced cardiac dysfunction and beta-adrenergic blockade therapy for sepsis. J Intensive Care. 2017;5:22. 28270914 10.1186/s40560-017-0215-2 PMC5335779

[40] Pavlov VA, Parrish WR, Rosas-Ballina M, Ochani M, Puerta M, Ochani K, et al. Brain acetylcholinesterase activity controls systemic cytokine levels through the cholinergic anti-inflammatory pathway. Brain Behav Immun. 2009;23(1):41–5. 18639629 10.1016/j.bbi.2008.06.011 PMC4533839

[41] Li G, Zhou CL, Zhou QS, Zou HD. Galantamine protects against lipopolysaccharide-induced acute lung injury in rats. Braz J Med Biol Res. 2016;49(2): e5008. 26648090 10.1590/1414-431x20155008 PMC4712483

[42] Hofer S, Eisenbach C, Lukic IK, Schneider L, Bode K, Brueckmann M, et al. Pharmacologic cholinesterase inhibition improves survival in experimental sepsis. Crit Care Med. 2008;36(2):404–8. 18091537 10.1097/01.CCM.0B013E31816208B3

[43] Danielski LG, Giustina AD, Badawy M, Barichello T, Quevedo J, Dal-Pizzol F, et al. Brain barrier breakdown as a cause and consequence of neuroinflammation in sepsis. Mol Neurobiol. 2018;55(2):1045–53. 28092082 10.1007/s12035-016-0356-7

[44] Borovikova LV, Ivanova S, Zhang M, Yang H, Botchkina GI, Watkins LR, et al. Vagus nerve stimulation attenuates the systemic inflammatory response to endotoxin. Nature. 2000;405(6785):458–62. 10839541 10.1038/35013070

[45] Huston JM, Gallowitsch-Puerta M, Ochani M, Ochani K, Yuan R, Rosas-Ballina M, et al. Transcutaneous vagus nerve stimulation reduces serum high mobility group box 1 levels and improves survival in murine sepsis. Crit Care Med. 2007;35(12):2762–8. 17901837 10.1097/01.CCM.0000288102.15975.BA

[46] Kohoutova M, Horak J, Jarkovska D, Martinkova V, Tegl V, Nalos L, et al. Vagus nerve stimulation attenuates multiple organ dysfunction in resuscitated porcine progressive sepsis. Crit Care Med. 2019;47(6):e461–9. 30908312 10.1097/CCM.0000000000003714

[47] Lankadeva YR, Booth LC, Kosaka J, Evans RG, Quintin L, Bellomo R, et al. Clonidine restores pressor responsiveness to phenylephrine and angiotensin ii in ovine sepsis. Crit Care Med. 2015;43(7):e221–9. 25860204 10.1097/CCM.0000000000000963

[48] Julien C, Orea V, Quintin L, Piriou V, Barres C. Renal sympathetic nerve activity and vascular reactivity to phenylephrine after lipopolysaccharide administration in conscious rats. Physiol Rep. 2017;5(4): e13139. 28242823 10.14814/phy2.13139 PMC5328774

[49] Coote JH. Landmarks in understanding the central nervous control of the cardiovascular system. Exp Physiol. 2007;92(1):3–18. 17030558 10.1113/expphysiol.2006.035378

[50] Savic B, Murphy D, Japundzic-Zigon N. The paraventricular nucleus of the hypothalamus in control of blood pressure and blood pressure variability. Front Physiol. 2022;13: 858941. 35370790 10.3389/fphys.2022.858941 PMC8966844

[51] Qin M, Gao Y, Guo S, Lu X, Zhao Q, Ge Z, et al. Establishment and evaluation of animal models of sepsis-associated encephalopathy. World J Emerg Med. 2023;14(5):349–53. 37908801 10.5847/wjem.j.1920-8642.2023.088 PMC10613796

[52] Han TH, Lee HW, Kang EA, Song MS, Lee SY, Ryu PD. Microglial activation induced by LPS mediates excitation of neurons in the hypothalamic paraventricular nucleus projecting to the rostral ventrolateral medulla. BMB Rep. 2021;54(12):620–5. 34814975 10.5483/BMBRep.2021.54.12.105 PMC8728541

