# Supplementary material for: Impact of sympathetic hyperactivity induced by brain microglial activation on organ damage in sepsis with chronic kidney disease
Source: J Intensive Care. 2024 Sep 2;12:31. doi: 10.1186/s40560-024-00742-2 (PMC11367766; doi:10.1186/s40560-024-00742-2)
Supplement: Supplementary file 1 — Additional file 1 [file 40560_2024_742_MOESM1_ESM.docx]

**Additional file 1: Supplemental Methods and Supplemental References.**

**Supplemental methods**

*1. Telemetry implantation*

We used the TA11 CTA-F40 telemetry systems (Data Sciences International, Saint Paul, MN, USA) to measure the electrocardiogram. 10-week-old rats were anesthetized with 2% isoflurane inhalation, and two electrodes and a telemeter were implanted subcutaneously. Briefly, using surgical scissors, make a 2.5 to 3.0 cm median incision on the dorsal aspect of the back between the two scapulae. Use blunt dissection to create a subcutaneous pocket along the dorsal flank on one side of the incision. Ensure that the pocket is sized appropriately for the type of transmitter being implanted. Place the transmitter in the pocket with the leads facing caudally. A 5 mm incision is then made in the bilateral thoracic region to create a subcutaneous tunnel between the dorsal subcutaneous pockets. The two electrodes of the transmitter are passed subcutaneously and each electrode is attached to the bilateral pectoral muscles. Finally, the skin is sutured.

*2. 5/6 nephrectomy*

Under 2.5% isoflurane anesthesia, with depth of anesthesia maintained by isoflurane inhalation (2.0% - 2.5%), the left kidney was exposed and partially infarcted by ligation of the anterior renal branch of its main artery followed by right nephrectomy. The skin was then closed with sutures and the animals were returned to their individual cages.

*3. Cecal ligation and puncture*

Rats were anesthetized with 2.0% - 2.5% isoflurane and a 2 cm incision was made in the ventral midline. The cecum was exposed and ligated distal to the ileocecal valve to avoid bowel obstruction, punctured once with an 18-gauge needle, gently squeezed to expel a small amount of stool, and returned to the abdominal cavity. The abdominal incision was closed in layers, and the animals received a subcutaneous dose of saline (3 ml/100 g) immediately after CLP.

*4. Renal function*

Urinary albumin and creatinine concentrations were measured by turbidimetric immunoassay and enzyme method, respectively (SRL Inc.), and the urinary albumin: creatinine ratio was calculated. CCr was calculated according to the following equation, as previously described [1]: CCr (mL/min/kg) = [urinary creatinine (mg/dL) × urine volume (mL)/plasma creatinine (mg/dL)] × [1000/body weight (g)] × [1/1440 (min)].

*5. Urine volume, water and food intake, and body weight*

Urine volume, water intake, food intake, and body weight were evaluated over a 24-hour period using a metabolic cage in each experimental period.

*6. Measurement of blood pressure and urinary norepinephrine*

Systolic blood pressure was measured in conscious rats by tail-cuff plethysmography (BP-98A; Softron Corp, Tokyo, Japan). Rats were warmed for 10 minutes before blood pressure measurement. The incubator was warmed to 37°C. Each value used in the analysis was an average of 5 blood pressure measurements obtained from each rat. We calculated 24-hour urinary norepinephrine (uNE) excretion using high-performance liquid chromatography as an indicator of sympathetic nerve activity (SRL Inc., Tokyo, Japan) as described previously [2, 3].

*7. Echocardiography*

Cardiac function was assessed by echocardiography. Serial M-mode echocardiography was performed on rats under light sodium pentobarbital anesthesia with spontaneous respiration using an SSD5000 (Aloka, Tokyo, Japan) equipped with a dynamically focused 10-MHz linear array transducer. M-mode tracings were recorded from the short-axis view at the level of the papillary muscle. Left ventricular (LV) end-diastolic diameter (LVDD) and LV end-systolic diameter (LVSD) were measured. Fractional shortening (FS) was calculated using the following equation: FS (%) = (LVDD - LVSD)/LVDD × 100.

*8. Blood sample analysis*

Plasma blood urea nitrogen (BUN), creatinine, total bilirubin, and platelet count were measured in blood samples collected from the left ventricle using a standard EDTA-containing syringe within minutes after the rats were injected with an overdose of sodium pentobarbital. Plasma obtained by centrifugation at 6000 rpm for 10 min was used for the above measurements (SRL Inc., Tokyo, Japan).

*9. Immunohistochemistry*

Rats were deeply anesthetized with an overdose of sodium pentobarbital and perfused with 0.9% cold saline (250 mL) followed by 4% formaldehyde (200 mL) through the left ventricle. The brain was removed and postfixed in 4% paraformaldehyde overnight at room temperature and then placed in 30% sucrose. The brains were embedded in O.C.T. compound (Tissue-Tek O.C.T. compound; Sakura Finetek Japan Co., Ltd., Tokyo, Japan) and coronally sectioned at 30 μm on a cryostat for free-float staining. Sections were incubated with 0.2% Triton X-100 and 3% bovine serum albumin.

For immunostaining of the paraventricular nucleus of the hypothalamus (PVN), sections were incubated with rabbit anti-Iba-1 antibody (1:1000; FUJIFILM Wako Pure Chemical Corporation, Osaka, Japan) overnight at 4°C. After washing, fluorescein (FITC)-conjugated donkey anti-rabbit IgG (1:300; Jackson ImmunoResearch Inc, PA, USA) was used for 12 hours at 4°C. Nuclear counterstaining was performed with a fluorescence-preserving mounting medium containing 4',6-diamidino-2-phenylindole (DAPI) (Vector, CA, USA). Using a NIKON A1 confocal laser microscope (20× eyepiece and 40× objective), we performed a detailed morphological evaluation of microglia within the hypothalamic fields in each animal.

For c-Fos immunostaining, sections including the PVN were incubated with rabbit anti-c-Fos antibody (1:500; EnCor Biotechnology Inc., FL, USA) for 60 minutes at room temperature. After washing, the sections were incubated with anti-rabbit secondary antibody (1:200; Alexa Fluor 488, abcam) for 60 minutes at room temperature. The number of c-Fos-positive cells in the PVN was counted using a NIKON A1 confocal laser microscope (20× eyepiece and 20× objective).

*10. Osmotic minipump implantation*

Under pentobarbital anesthesia and using a stereotaxic frame, a 23-gauge right-angled stainless-steel cannula was implanted into the right lateral cerebral ventricle and fixed to the rat skull with acrylic cement. The cannula was placed 1.0 mm posterior and 1.5 mm lateral to the bregma. The lower end (shorter arm) of the cannula was at a depth of 3.7 mm from the dura, and the upper end (longer arm) was connected to an osmotic minipump (ALZET, model 2002, Muromachi Kikai Co., Ltd., Tokyo, Japan) for chronic intracerebroventricular infusion at 0.5μL/h for 2 days. The pumps were filled with aCSF alone or in combination with minocycline (10 mg/ml) and implanted subcutaneously on the back of the rats. The composition of the aCSF was (in mmol/l) 121 NaCl, 3.4 KCl, 1.2 MgCl2, 0.6 NaH2PO4, 29 NaHCO3, and 3.4 glucose. The pH was 7.4 and the osmolarity was 296 mosmol/kg H2O.

**Supplemental References**

1. Liu IM, Tzeng TF, Liou SS, Chang CJ. Beneficial effect of traditional chinese medicinal formula danggui-shaoyao-san on advanced glycation end-product-mediated renal injury in streptozotocin-diabetic rats. Evid Based Complement Alternat Med. 2012;2012:140103.

2. Nishihara M, Hirooka Y, Kishi T, Sunagawa K. Different role of oxidative stress in paraventricular nucleus and rostral ventrolateral medulla in cardiovascular regulation in awake spontaneously hypertensive rats. J Hypertens. 2012;30(9):1758—65.

3. Nishihara M, Hirooka Y, Sunagawa K. Combining irbesartan and trichlormethiazide enhances blood pressure reduction via inhibition of sympathetic activity without adverse effects on metabolism in hypertensive rats with metabolic syndrome. Clin Exp Hypertens. 2015;37(1):33—8.
