# Supplementary material for: Impact of sympathetic hyperactivity induced by brain microglial activation on organ damage in sepsis with chronic kidney disease
Source: J Intensive Care. 2024 Sep 2;12:31. doi: 10.1186/s40560-024-00742-2 (PMC11367766; doi:10.1186/s40560-024-00742-2)
Supplement: Supplementary file 2 — Additional file 2. [file 40560_2024_742_MOESM2_ESM.docx]

**Additional file 2: Supplemental Figures 1-9 and Supplemental Tables 1-4.**

Figure S1: Temporal changes in rectal temperature

Figure S2: Localization of the paraventricular nucleus in the hypothalamus

Figure S3: Assessment of microglial and neuronal activation in the paraventricular nucleus

Figure S4: Effect of intracerebroventricular minocycline administration on microglial and neuronal activation

Figure S5: Effects of minocycline on blood pressure and heart rate after sepsis

Figure S6: Time course of changes in low/high frequency ratio in minocycline-treated septic rats

Figure S7: Influence of minocycline on sepsis response in renal disease-free rats

Figure S8: Effects of minocycline on blood parameters after sepsis in renal disease-free rats

Figure S9: Influence of intracerebroventricular minocycline on sympathetic nervous system activation after sepsis in renal disease-free rats

Table S1: Characteristics of chronic kidney disease induced by 5/6 nephrectomy

Table S2: Echocardiographic effects of 5/6 nephrectomy and cecal ligation and puncture in rats

Table S3: Physiological parameter effects of 5/6 nephrectomy and cecal ligation and puncture in rats

Table S4: Post hoc power analysis of an intracerebroventricular microglial inhibitor in a CKD-complicated sepsis rat model

**Supplemental Figure 1**

**
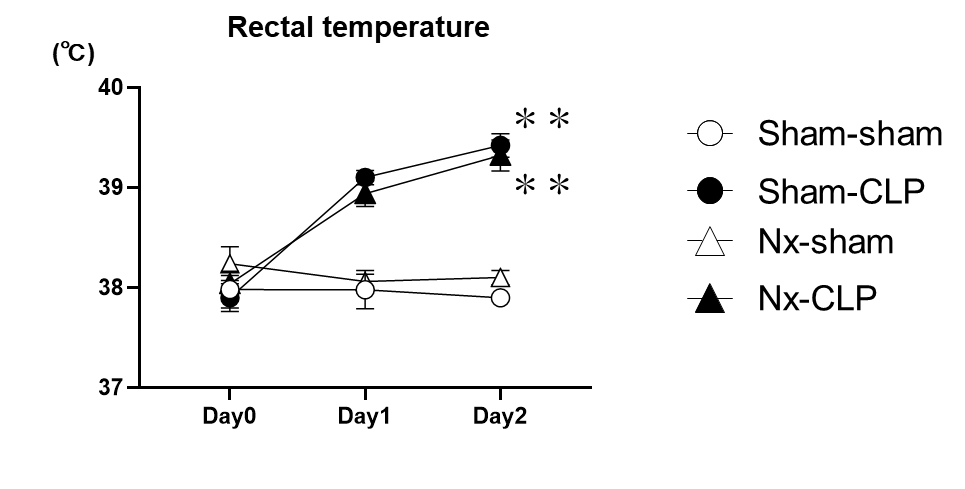
**

**Supplemental Figure 1 legends.** Time course of changes in rectal temperature after cecal ligation and puncture (CLP) or sham surgery 4 weeks after 5/6 nephrectomy (Nx) or sham surgery. Values are mean ± SEM; n = 5 for each group. Two-way ANOVA with multiple comparisons, ** p < 0.01, compared to each control (i.e., comparison between sham-CLP and sham-sham groups).

**Supplemental Figure 2**

**Location of paraventricular nucleus of the hypothalamus (PVN)**

**
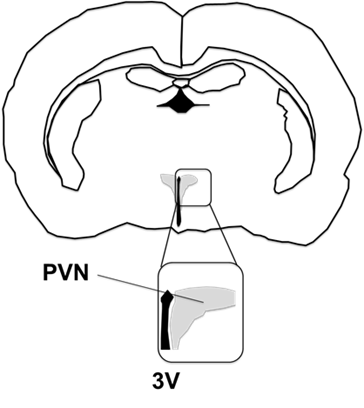
**

**Supplemental Figure 2 legends.** The figure shows a coronal section of the rat brain. The grey areas represent the bilateral paraventricular nucleus of the hypothalamus (PVN). Regions enclosed by squares represent areas stained with c-fox immunohistochemistry. 3V indicates the third ventricle.

**Supplemental Figure 3**

**
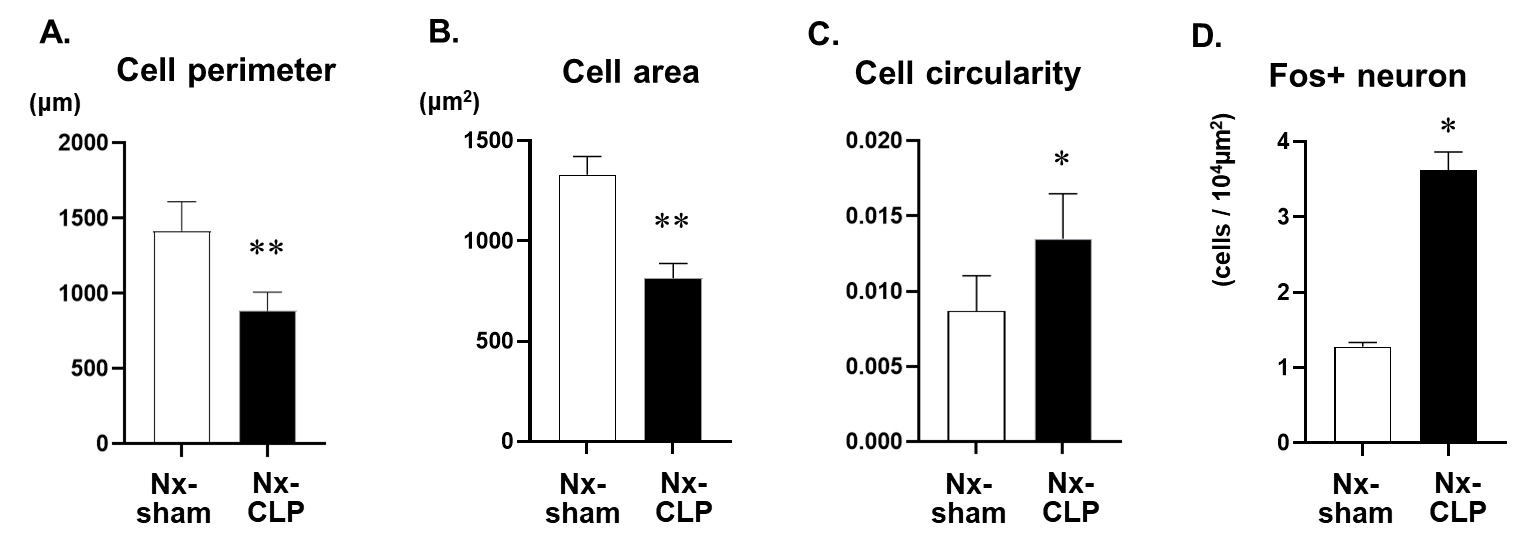
**

**Supplemental Figure 3 legends.** Effects of CLP on microglia and neuronal activity in the PVN of Nx rats. Four weeks after Nx surgery, CLP or sham surgery was performed (Nx-CLP group and Nx-sham group). Immunohistochemical staining of macrophages with anti-Iba-1 antibody in PVN was performed in Nx-sham group and Nx-CLP group. Figures (A), (B), and (C) show cell perimeter, cell area, and cell circularity of macrophages, respectively. The Nx-CLP group had significantly smaller cell perimeter and cell area and significantly larger cell circularity than the Nx-sham group. Graph (D) shows the number of c-Fos-positive neurons calculated from the results of immunohistochemical staining of neurons in the PVN using anti-c-Fos antibody in the Nx-sham and Nx-CLP groups. There were significantly more c-Fos-positive neurons in the Nx-CLP group than in the Nx-sham group. Values are mean ± SEM; n = 5 for each group. * p < 0.05, and ** p < 0.01, compared with Nx-sham rats.

**Supplemental Figure 4**

**
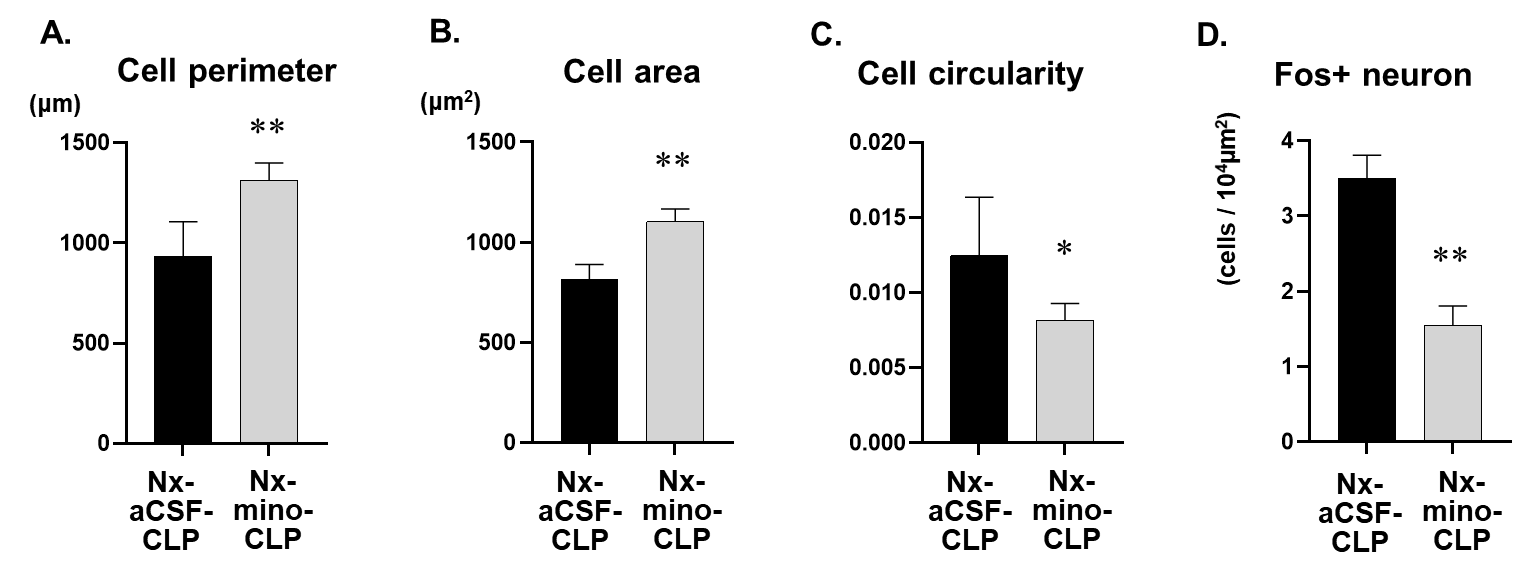
**

**Supplemental Figure 4 legends.** Intracerebroventricular minocycline effects on microgia and neuronal activity in the paraventricular nucleus of the hypothalamus (PVN) in cecal ligation and puncture (CLP)-induced 5/6 nephrectomy (Nx) rats. Four weeks after Nx surgery, minocycline (mino) or artificial cerebrospinal fluid (aCSF) was administered intracerebroventricularly before CLP surgery (Nx-mino-CLP group and Nx-aCSF-CLP group). Immunohistochemical staining of PVN microglia with anti-Iba-1 antibody was performed. The Nx-mino-CLP group showed significantly larger macrophage perimeter and area and smaller circularity compared with the Nx-aCSF-CLP group. Immunohistochemical staining of PVN neurons with anti-c-Fos antibody showed significantly fewer c-Fos-positive neurons in the Nx-mino-CLP group than in the Nx-aCSF-CLP group. Values represent mean ± SEM; n = 5 per group. * p < 0.05, ** p < 0.01 versus Nx-aCSF-CLP group. SEM = standard error of the mean, Iba-1 = ionized calcium-binding adaptor molecule 1.

**Supplemental Figure 5**


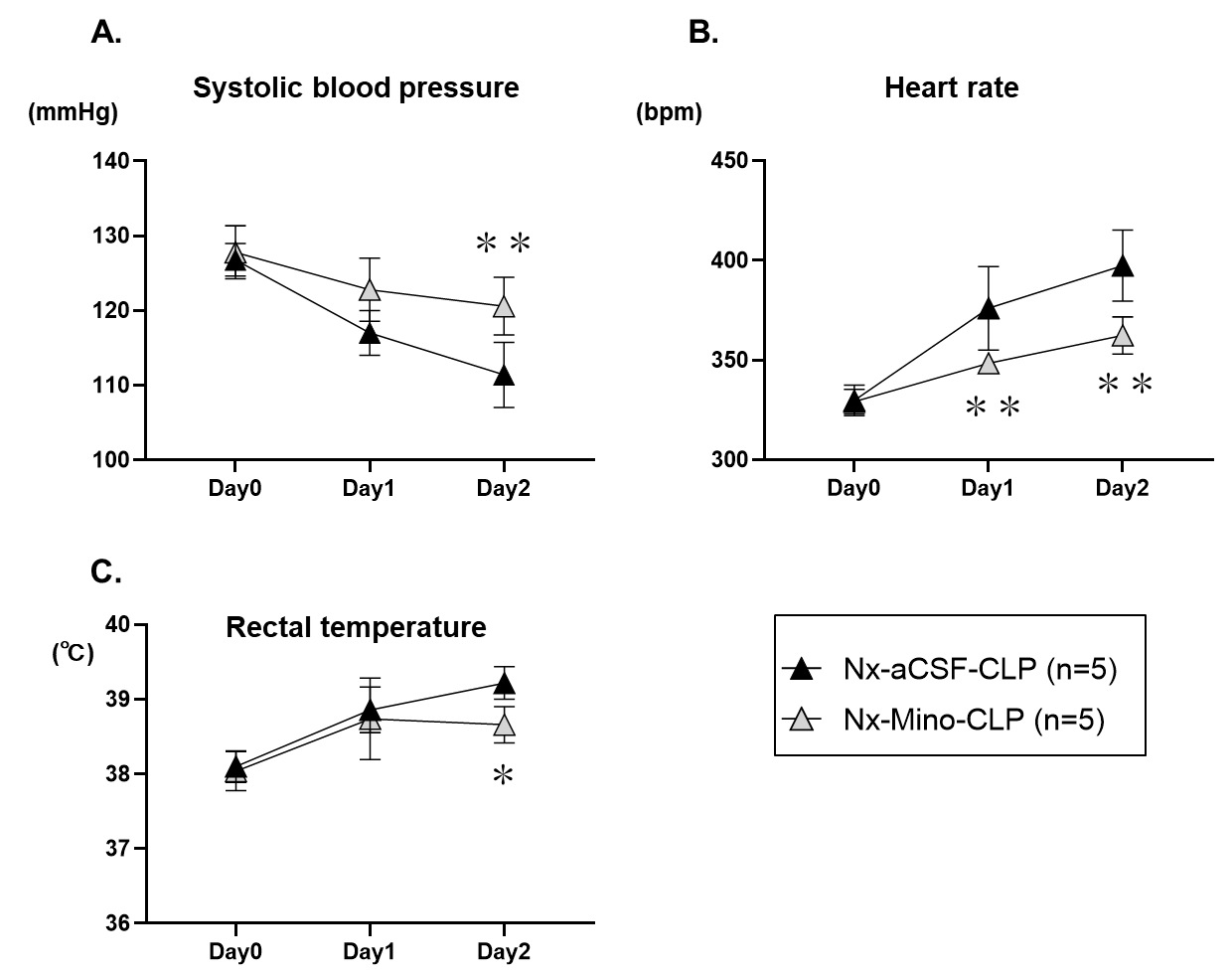


**Supplemental Figure 5 legends.** Four weeks after 5/6 nephrectomy (Nx) surgery, minocycline or artificial cerebrospinal fluid (aCSF) was administered intracerebroventricularly followed by cecal ligation and puncture (CLP) surgery (Nx-mino-CLP group and Nx-aCSF-CLP group, respectively). This is shown in the graphs showing the time course of changes in systolic blood pressure (*A*), heart rate (B) and rectal temperature in each group. Values are mean ± SEM; n = 5 for each group. Two-way ANOVA with multiple comparisons, * p < 0.05, ** p < 0.01, vs. Nx CLP with aCSF administration.

**Supplemental Figure 6**


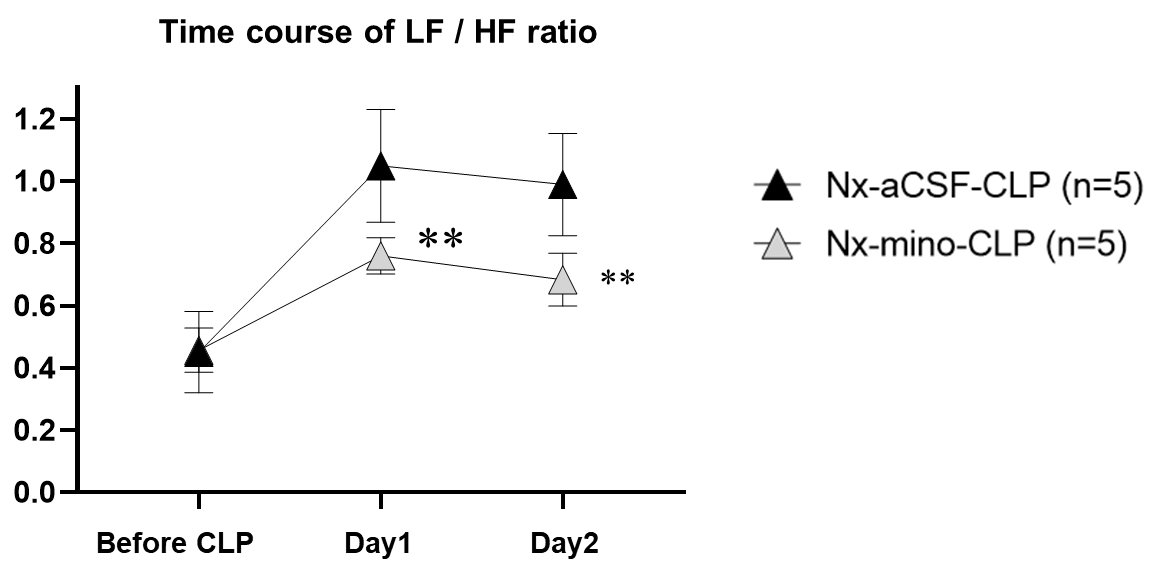


**Supplemental Figure 6 legends.** Four weeks after sham surgery for 5/6 nephrectomy, minocycline or aCSF was administered intracerebroventricularly followed by CLP surgery (Nx-aCSF-CLP or Nx-mino-CLP group, respectively). The graph shows the time course (day1, day2) of the LF/HF ratio in each group before and after CLP surgery. Data are expressed as mean ± SEM. Two-way ANOVA with multiple comparisons, ** p < 0.01, compared with Nx-aCSF-CLP group.


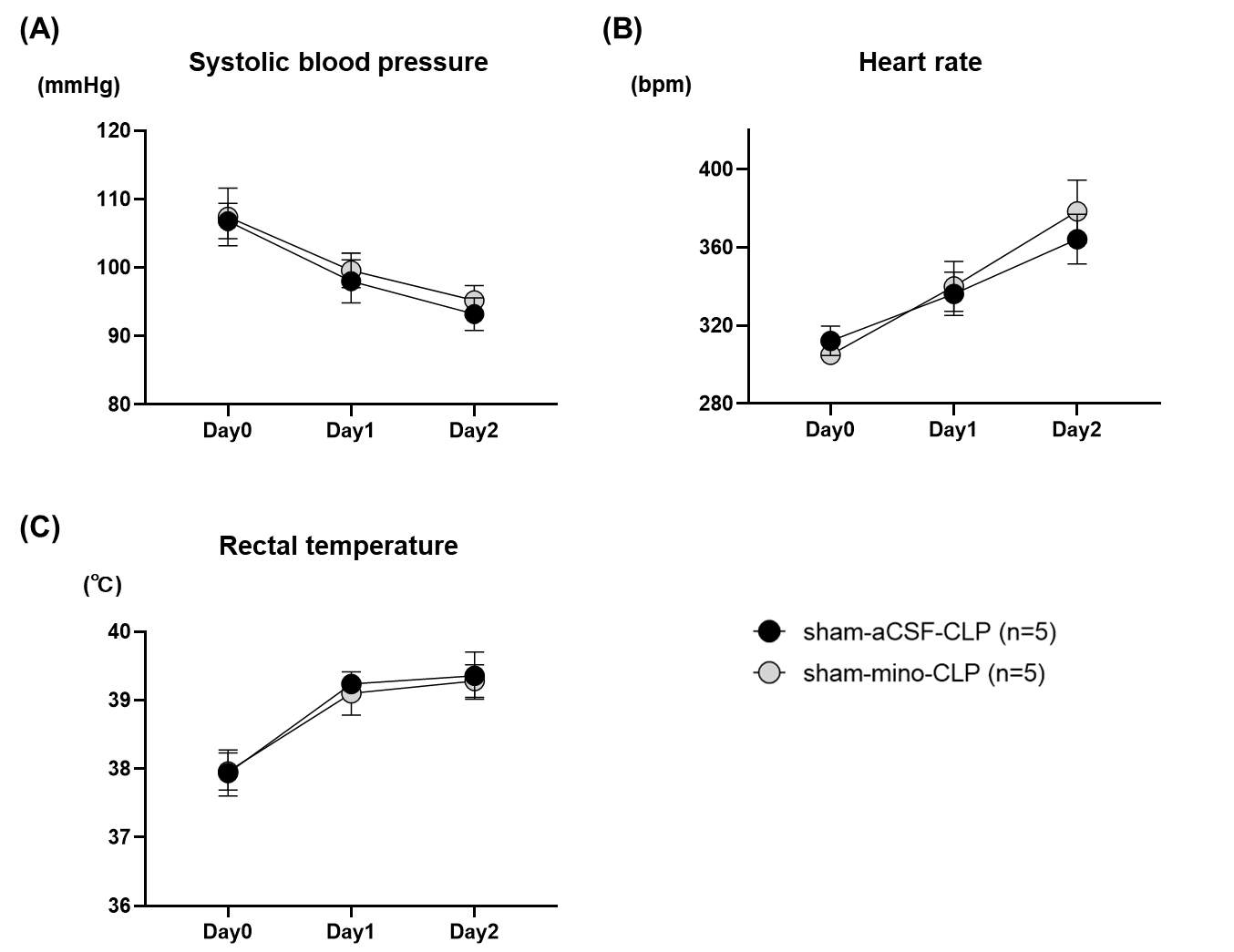
 **Supplemental Figure 7**

**Supplemental Figure 7 legends.** Four weeks after sham surgery for 5/6 nephrectomy, minocycline or aCSF was administered intracerebroventricularly, followed by CLP surgery. This is shown in the graphs showing the time course of changes in systolic blood pressure (A), heart rate (B), and rectal temperature (C), respectively. Values are mean ± SEM; n=5 for each group. Two-way ANOVA for multiple comparisons. There was no statistically significant difference between the two groups.


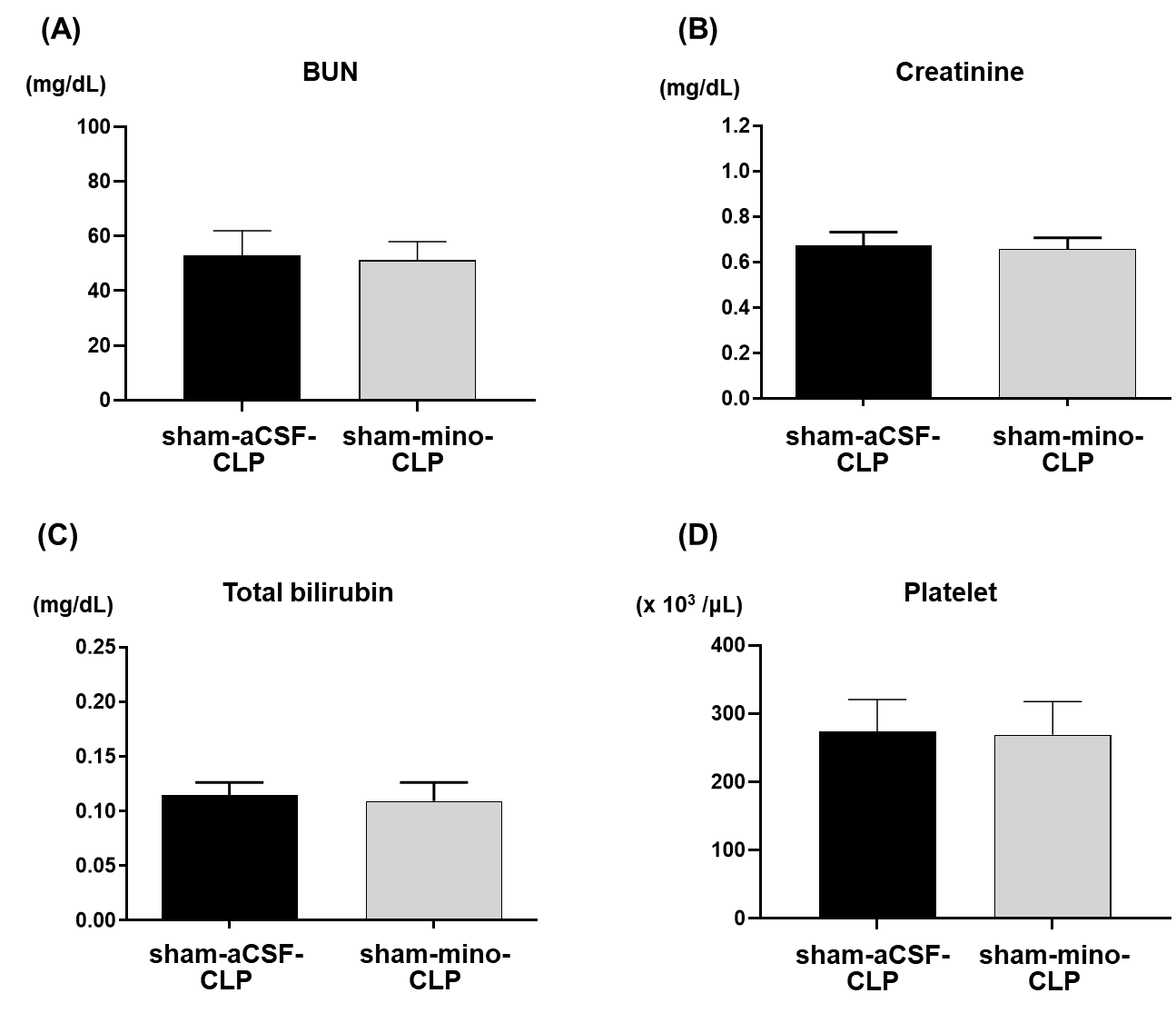
**Supplemental Figure 8**

**Supplemental Figure 8 legends.** Effect of intracerebroventricular minocycline on blood test results in CLP-Induced Non-CKD rats. At the fourth week after sham surgery for 5/6 nephrectomy, intracerebroventricular administration of minocycline (mino) or aCSF was initiated, followed by CLP surgery (sham-mino-CLP group and sham-aCSF-CLP group). Graphs (A)-(D) show the effects of CLP in both groups on blood test results. Data are expressed as mean ± SEM. Unpaired t-test. There was no statistically significant difference between the two groups (n=5 for each).

**Supplemental Figure 9**


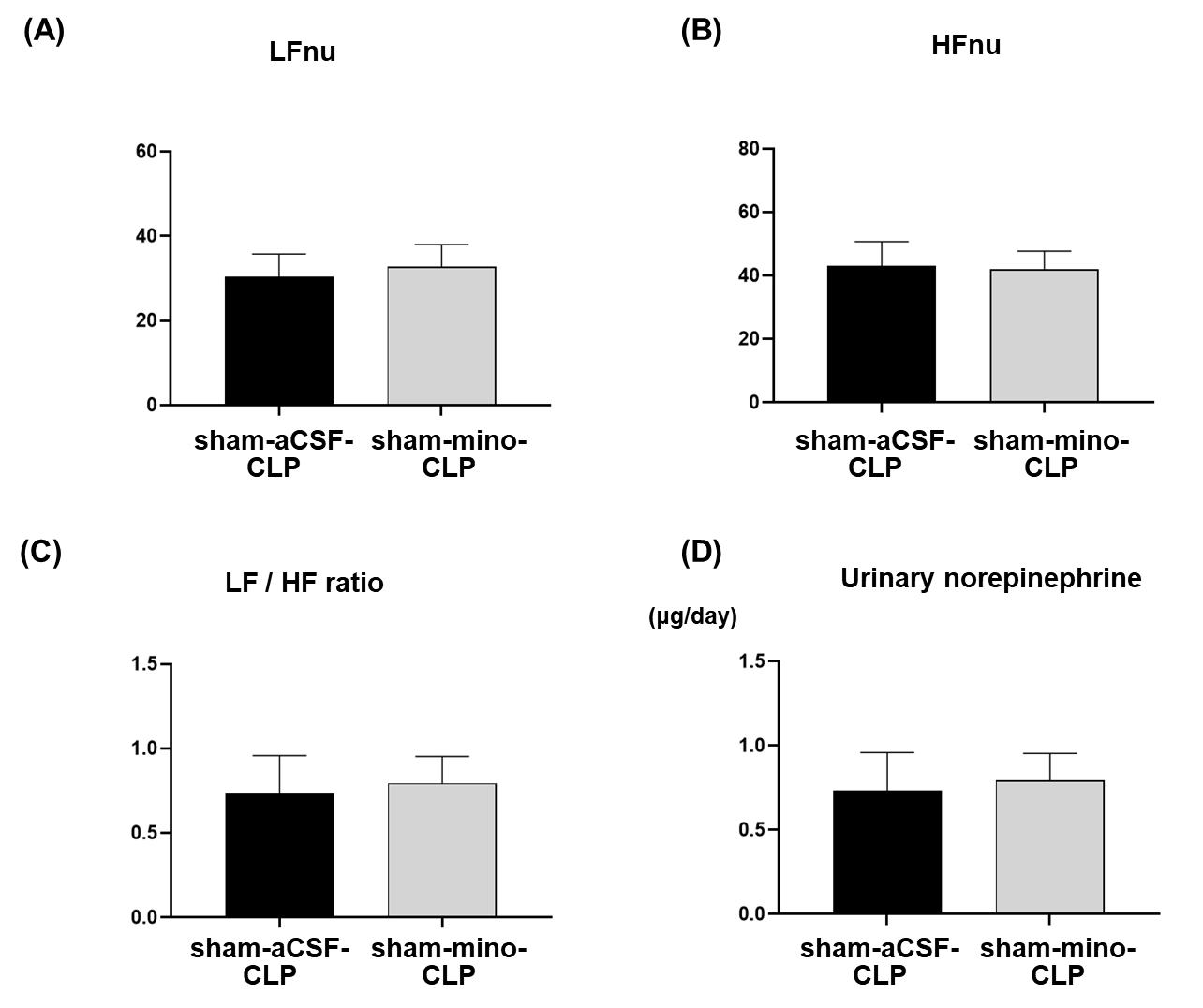


**Supplemental Figure 9 legends.** Effect of intracerebroventricular minocycline on sympathetic nervous system activity in CLP-induced Non-CKD rats. At the fourth week after sham surgery for 5/6 nephrectomy, intracerebroventricular administration of minocycline (mino) or aCSF was initiated, followed by CLP surgery (sham-mino-CLP group and sham-aCSF-CLP group). Graphs (A), (B), and (C) show the effects of CLP in sham-mino or sham-aCSF rats on low-frequency component (LFnu), high-frequency component (HFnu), and LF/HF ratio obtained by analysis of heart rate data obtained by electrocardiographic telemetry. Graph (D) shows the effects of CLP in sham-mino or sham-aCSF rats on urinary norepinephrine excretion based on a 24-hour urine collection. Data are expressed as mean ± SEM. Unpaired t-test. There was no statistically significant difference between the two groups (n=5 for each).

**Supplemental table 1**

Characteristics of 5/6 nephrectomy (Nx) induced chronic kidney disease in rats

|  | Control rats | Nx rats |
| --- | --- | --- |
| Systolic blood pressure (mm Hg) | 109±1 | 128±2** |
| Heart rate (per minute) | 311±5 | 344±6** |
| Plasma creatinine concentration (mg/dL) | 0.35±0.03 | 0.57±0.04** |
| Creatinine clearance (mL/min/kg) | 0.83±0.03 | 0.49±0.04** |
| Urinary albumin to creatinine ratio (mg/g・Cre) | 0.08±0.02 | 0.20±0.04* |
| Urinary norepinephrine (µg/day) | 1.1±0.1 | 1.5±0.1* |
| Heart rate variability analysis |  |  |
| LFnu | 21.3±1.2 | 31.3±2.3** |
| HFnu | 65.1±5.2 | 67.1±6.0 |
| LF/HF ratio | 0.34±0.03 | 0.47±0.02** |
| Echocardiography |  |  |
| LVDd (mm) | 7.5±0.1 | 7.4±0.2 |
| LVDs (mm) | 2.9±0.1 | 2.4±0.0* |
| LV wall thickness (mm) | 1.4±0.0 | 1.6±0.0** |
| % FS (%) | 62.2±1.4 | 66.9±0.6* |
| Inferior vena cava (mm) | 4.5±0.4 | 3.1±0.3* |
| Collapsibility index (%) | 22.5±3.4 | 31.8±3.0† |
| Physiological parameters |  |  |
| Body weight (g) | 475±8 | 454±3* |
| Urine volume (ml) | 18±2 | 27±4* |
| Water intake (ml) | 37±2 | 53±4* |
| Food intake (g) | 20±1 | 22±2 |

Abbreviations are as follows. LVDd; LV end-diastolic diameter, LVDs; LV end-systolic diameter, and %FS; percent fractional shortening. Values are mean ± SEM. * p < 0.05 and ** p < 0.01, compared with sham operation of Nx, † 0.05 < p < 0.1, compared with sham operation of Nx.

**Supplemental table 2**

Effects of 5/6 nephrectomy (Nx) and cecal ligation and puncture (CLP) in rats on echocardiography

|  | Sham-sham rats (n=6) | Sham-CLP rats (n=6) | Nx-sham rats (n=6) | Nx-CLP rats (n=6) |
| --- | --- | --- | --- | --- |
| LVDd (mm) | 7.2±0.1 | 6.3±0.2** | 7.1±0.1 | 5.9±0.3** |
| LVDs (mm) | 2.5±0.1 | 1.8±0.1** | 2.1±0.1 | 1.5±0.0** |
| IVS (mm) | 1.3±0.0 | 1.3±0.0 | 1.6±0.0 | 1.5±0.0 |
| LVPW (mm) | 1.4±0.0 | 1.4±0.0 | 1.7±0.0 | 1.6±0.0 |
| %FS (%) | 65.4±1.6 | 70.6±1.9 | 70.8±0.9 | 74.6± 0.7 |
| IVCD (mm) | 4.8±0.3 | 2.5±0.1** | 3.1±0.3 | 1.9±0.1** |

Abbreviations are as follows. LVDd; LV end-diastolic diameter, LVDs; LV end-systolic diameter, IVS; interventricular septum thickness, LVPW; left ventricular posterior wall thickness, %FS; percent fractional shortening, and IVCD; inferior vena cava diameter. Values are mean ± SEM; n, number of rats in each group. **p < 0.01, compared with sham operation of each CLP group.

**Supplemental table 3**

|  | Sham-sham rats (n=5) | Sham-CLP rats (n=5) | Nx-sham rats (n=5) | Nx-CLP rats (n=5) |
| --- | --- | --- | --- | --- |
| Body weight (g) |  |  |  |  |
| Nx, 4 week | 475±8 | 474±4 | 454±3* | 457±3 |
| CLP, 2 day | 471±5 | 458±6 | 448±5 | 434±5† |
| Urine volume (ml) |  |  |  |  |
| Nx, 4 week | 18±2 | 20±1 | 27±4* | 29±1 |
| CLP, 2 day | 17±2 | 12±1 | 24±3 | 12±2^#^ |
| Water intake (ml) |  |  |  |  |
| Nx, 4 week | 37±2 | 36±2 | 53±4* | 53±3 |
| CLP, 2 day | 37±3 | 22±6† | 47±4 | 29±3^#^ |
| Food intake (g) |  |  |  |  |
| Nx, 4 week | 20±1 | 19±1 | 22±2 | 22±1 |
| CLP, 2 day | 19±0 | 5±13^#^ | 21±1 | 4±3^#^ |

Effects of 5/6 nephrectomy (Nx) and cecal ligation and puncture (CLP) in rats on physiological parameters

Values are mean ± SEM; n, number of rats in each group. * p < 0.05, compared with sham operation of Nx, # p < 0.05, compared with sham operation of each CLP, † 0.05 < p < 0.1, compared with sham operation of each CLP.

**Supplemental table 4**

Post hoc statistical power analysis of the effects of intracerebroventricular administration of a microglial inhibitor in a rat model of CKD complicated sepsis

| Variables | Nx-aCSF-CLP rats (n=5) | Nx-mino-CLP rats (n=5) | p | Difference between the means of the two groups | Common SD of the two groups | post hoc statistical power |
| --- | --- | --- | --- | --- | --- | --- |
| Hemodynamics |  |  |  |  |  |  |
| Systolic blood pressure (mm Hg) | 111 ± 2 | 120 ± 2 | 0.02 | 9.20 | 4.09 | 0.95 |
| Heart rate (per minute) | 397 ± 8 | 362 ± 4 | 0.008 | 35.00 | 13.55 | 0.98 |
| Blood test |  |  |  |  |  |  |
| Blood urea nitrogen  (mg/dL) | 79 ± 3 | 61 ± 5 | 0.02 | 17.22 | 9.08 | 0.85 |
| Plasma creatinine concentration (mg/dL) | 0.95 ± 0.09 | 0.72 ± 0.03 | 0.03 | 0.23 | 0.12 | 0.83 |
| Total billirubin (mg/dL) | 0.20 ± 0.02 | 0.13 ± 0.01 | 0.02 | 0.07 | 0.04 | 0.86 |
| Platelet count (x 10^3^ /μL) | 198 ± 25 | 309 ± 33 | 0.03 | 110.40 | 68.42 | 0.76 |
| Heart rate variability analysis |  |  |  |  |  |  |
| LFnu | 43.6 ± 1.7 | 35.8 ± 2.7 | 0.04 | 7.86 | 4.98 | 0.70 |
| HFnu | 41.5 ± 1.5 | 55.0 ± 3.4 | 0.007 | 13.53 | 5.54 | 0.97 |
| LF/HF ratio | 1.05 ± 0.08 | 0.76 ± 0.03 | 0.008 | 0.29 | 0.12 | 0.97 |
| Urinary norepinephrine  (µg/day) | 3.7 ± 0.2 | 2.7 ± 0.2 | 0.07 | 1.00 | 0.44 | 0.95 |

Abbreviations are as follows. CLP; cecal ligation and puncture, Nx; 5/6 nephrectomy, aCSF; artificial cerebrospinal fluid, and mino; minocycline, LFnu; low frequency power in normalized units, HFnu; high frequency power in normalized units, SD; standard deviation. Values are mean ± SEM.
